# Platelet Membrane Nanocarriers Cascade Targeting Delivery System to Improve Myocardial Remodeling Post Myocardial Ischemia–Reperfusion Injury

**DOI:** 10.1002/advs.202308727

**Published:** 2024-02-12

**Authors:** Xuan Xu, Mingxi Li, Fuchao Yu, Qin Wei, Yang Liu, Jiayi Tong, Fang Yang

**Affiliations:** ^1^ Department of Cardiology Zhongda Hospital Affiliated to Southeast University 87, Dingjiaqiao Nanjing 210009 P. R. China; ^2^ State Key Laboratory of Digital Medical Engineering Jiangsu Key Laboratory for Biomaterials and Devices School of Biological Sciences and Medical Engineering Southeast University Nanjing 210096 P. R. China

**Keywords:** cardioprotection, FTY720, L‐arginine, multitarget strategies, platelet membrane, reperfusion inflammatory

## Abstract

Although treatments for myocardial infarction have advanced significantly, the global mortality due to ischemia and subsequent reperfusion injury remains high. Here, a platelet (PLT) membrane nanocarrier (PL720) that encapsulates L‐arginine and FTY720 to facilitate the cascade‐targeted delivery of these substances to the myocardial injury site and enable the controlled release of L‐arginine and FTY720 is developed. Such an innovative approach shows enhanced cardioprotection through multiple target strategies involved in ischemia–reperfusion injury and late reperfusion inflammation. During the ischemia–reperfusion phase, PL720 targets and accumulates in damaged coronary arteries. PL720 rapidly releases L‐arginine, stimulating endothelial cells to produce NO, thereby dilating blood vessels and promoting blood flow recovery, while FTY720's sustained release exerts anti‐apoptotic effects. During the late reperfusion inflammatory phase, PL720 is captured by circulating inflammatory monocytes and transported into a deeper ischemic myocardial lesion. PL720 promotes macrophage polarization and accelerates the inflammatory repair. Furthermore, the issue of bradycardia associated with the clinical use of FTY720 is innovatively relieved. Therefore, PL720 is a vascular injury and inflammation dual targeting strategy, exhibiting significant potential for multi‐targeted therapy and clinical translation for cardiac injury.

## Graphical abstract

A nanocarrier encapsulated with FTY720 and L‐arginine in the platelet membrane is developed, resulting in multi‐targeting capability for myocardial ischemia–reperfusion injury lesions. The nanocarrier exert effects by promoting vasodilation, anti‐apoptosis, and anti‐inflammatory responses. The dual‐target triple therapy strategy successfully reverses the cardiac function impairment caused by ischemia–reperfusion injury.

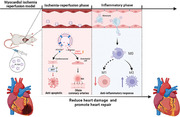

## 1 Introduction

Acute myocardial infarction (AMI) is a major cardiovascular disease and the primary cause of mortality worldwide.^[^ ^1^ ^]^ Following the occurrence of AMI, the most efficient and well‐established clinical treatment approach is to promptly restore blood flow and achieve reperfusion via percutaneous coronary intervention or thrombolysis. It aims to minimize myocardial ischemic damage, preserve the viable myocardium, and restrict infarct expansion.^[^ ^2^ ^]^ Nonetheless, the re‐establishment of blood flow to the ischemic myocardium during early reperfusion can paradoxically trigger oxidative stress and heighten inflammatory responses, resulting in myocardial cell injury, a phenomenon referred to as myocardial ischemia/reperfusion (MI/R) injury. This injury mechanism has the potential to contribute to up to 50% of the final infarct size. Even when the blood supply is restored by percutaneous coronary intervention, mortality and recurrence rates within the first year after acute infarction onset remain considerably elevated, posing a severe threat to patient survival and quality of life.^[^ ^3^ ^]^

The MI/R process involves intricate intercellular and intracellular interactions and molecular mechanisms encompassing the generation of oxidative stress, apoptosis, autophagy, cell pyroptosis, and the emergence of diverse inflammatory responses. During the initial phase of ischemia–reperfusion, the re‐establishment of blood flow prompts the rapid accumulation of reactive oxygen species (ROS) to induce apoptosis in cardiomyocytes.^[^ ^4^ ^]^ In the reperfusion process, cell apoptosis and inflammatory responses are assumed to play pivotal roles in the formation of infarct foci and subsequent myocardial remodeling. In particular, the balance between the inflammatory macrophages (M1)‐type pro‐inflammatory and reparative macrophage (M2)‐type anti‐inflammatory states is essential for clearing cellular debris from infarcted areas for effective remodeling. Delayed production of M2‐type macrophages may exacerbate adverse myocardial remodeling.^[^ ^5^ ^]^Therefore, modulation of inflammatory responses represents a crucial target for intervention in MI/R injury.

Sphingosine 1‐phosphate (S1P), a biologically active lysophospholipid, exerts its influence via five G‐protein‐coupled S1P receptors (S1PR1‐R5) and plays a significant role in the development and progression of acute infarction. It contributes to various processes such as protecting cardiomyocytes during MI/R, regulating immune cell migration and differentiation, and influencing myocardial remodeling.^[^ ^6^ ^]^ FTY720 (fingolimod) is an immunomodulatory drug approved by the Food and Drug Administration (FDA) in 2010 for clinical use in humans.^[^ ^7^ ^]^ It is a synthetic S1P analog that serves as an S1P receptor agonist for the treatment of multiple sclerosis and multiple organ transplantation. FTY720 promotes M2 phenotypic polarization in macrophages/microglia, thereby exhibiting anti‐inflammatory immunomodulatory effects. The major mechanism involves activation of the c‐Jun N‐terminal Kinase (JNK)/ Signal Transducer and Activator of Transcription 3 (STAT3) signaling pathway and subsequent inhibition of immune cell adhesion via reduced expression of adhesion molecules.^[^ ^8^ ^]^ Although FTY720‐activated cardiac S1P receptors have been demonstrated to significantly reduce cardiomyocyte apoptosis through the activation of the S1PR1‐ Protein Kinase B (AKT) signaling pathway,^[^ ^9^ ^]^ the anti‐inflammatory mechanism of FTY720 during MI/R remains poorly understood because it is a non‐selective agonist for all S1P receptors. It also functions as an antagonist of S1P receptors. Systemic administration of FTY720 may result in the irreversible internalization and degradation of S1PRs, leading to S1PRs‐dependent functional deficits.^[^ ^10^ ^]^ Furthermore, a dose‐dependent reduction in heart rate observed at 6 h following the initial FTY720 administration is associated with an elevated risk of sudden death among patients with AMI.^[^ ^11^ ^]^ Thus, the effective and targeted delivery of FTY720 with a slow release rate, while minimizing adverse effects on non‐target organs during drug release remains a significant challenge for MI/R injury treatment.

**Figure 1:**
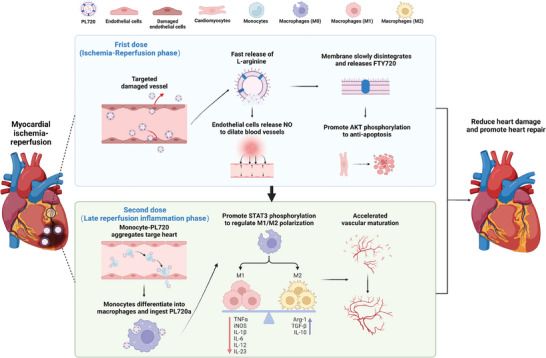
Schematic illustration of PL720 cascade targeted therapy for MI/R in mice. When PL720 is administered at ischemia‐ reperfusion phase, it effectively targets the site of injury. Upon reaching the lesion site, the encapsulated *L*‐arginine is released to facilitate the production of NO for timely vasodilation. Simultaneously, the slow‐released FTY720 within the injured myocardium activates the AKT pathway in cardiomyocytes to alleviate cardiomyocyte apoptosis. When administered at the late perfusion inflammation phase, the PL720 captured monocyte (monocyte‐PL720 aggregates) is recruited to the heart lesion. Recruited monocytes in the heart differentiate into macrophages, and subsequently, the macrophages phagocytize PL720. With the sustained release of FTY720, the STAT3 signaling pathway of macrophages is activated, thereby promoting macrophage polarization. This process reduces sprouting vessel degeneration caused by long M1 subtype macrophages while increasing M2 subtype macrophages to promote the maturation and quiescence of sprouting vessels.

MI/R interventions are divided into three distinct phases: ischemia, ischemia–reperfusion, and late reperfusion (including the inflammatory phase).^[^ ^4^ ^b]^ Maximum cardioprotection effect can be achieved by targeting two or more therapeutic windows. Herein, inspired by the recruitment of platelets (PLTs) and the formation of monocyte‐platelet aggregates following MI/R,^[^ ^12^ ^]^ we developed a platelet membrane‐encapsulated L‐arginine and FTY720 biomimetic nanocarrier (PL720) with a mean diameter of 200 nm. In our previous study, we demonstrated that L‐arginine encapsulated in platelet membranes prompted the rapid release of nitric oxide (NO) at the targeted site, leading to local blood vessel dilation and enhanced delivery efficiency.^[^ ^13^ ^]^ As shown in **Figure** **1** during the ischemic–reperfusion phase, the PL720 nanocarriers effectively targeted MI/R lesions because of the natural recognition activity of the platelet membrane. Utilizing the fast release of L‐arginine and delayed release of FTY720, PL720 nanocarriers can reduce cardiomyocyte apoptosis in the early post‐MI/R phase. Second, during the late reperfusion inflammatory phase, PL720 is recruited to inflammatory‐responsive monocytes to form monocyte‐PL720 aggregates and enter the damaged heart area. Promoting the STAT3 phosphorylation facilitates reprogramming of the inflammatory setting late into reperfusion. In summary, the fabricated PL720 can specifically target different time points in the MI/R setting (during ischemia–reperfusion and late reperfusion inflammation). It combines rapid blood restoration with cardiomyocyte anti‐apoptosis and immune modulation therapies to achieve additive cardioprotection. The high sensitivity to damaged vascular vessels and controlled release characteristics of PL720 is endowed with a synergistic multitarget therapeutic effect, which shows as a promising therapeutic strategy for MI/R in clinic in the future.

## 2 Results and Discussion

### 2.1 Size, Morphology, and Biological Function Characterization of PL720

**Figure 2:**
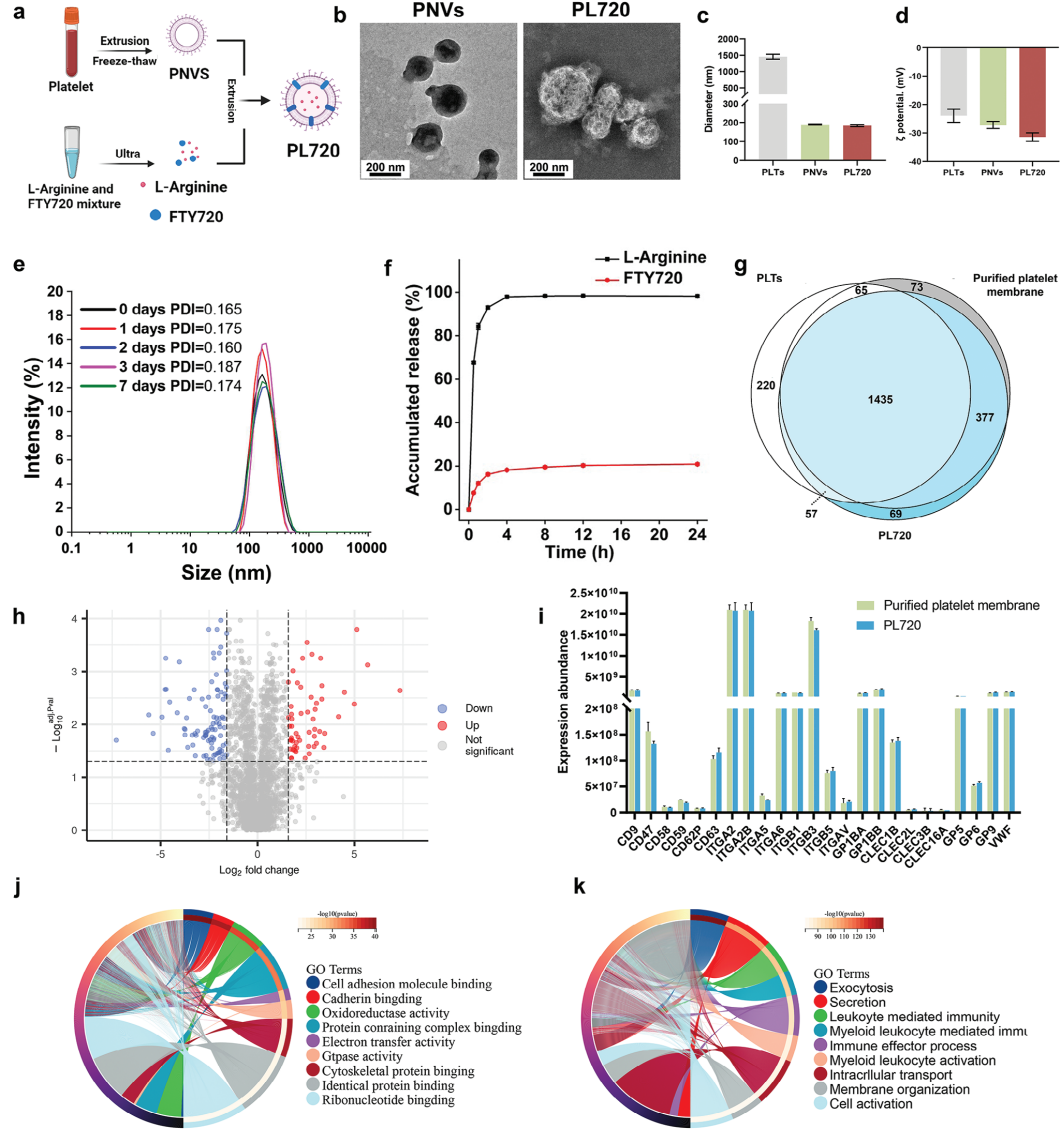
Preparation and characterization of PL720. a) Schematic diagram of PL720 fabrication. b) TEM image of PNVs and PL720. c) Mean diameter of PLTs, PNVs, and PL720 (*n* = 5). d) Zeta potential (ξ) analysis of PLTs, PNVs, and PL720 (*n* = 5). e) Polymer dispersity index (PDI) of PLTs, PMVs, and PL720 (*n* = 3). f. Release profiles of L‐arginine and FTY720 from PL720 within 24 h. g) Quantification of proteins in PLTs, purified platelet membrane, and PL720 identified by LC‐MS/MS. The Venn diagram illustrates the protein overlap among PLTs, purified platelet membrane, and PL720. h) Volcano plot displaying differential proteins between purified platelet membrane and PL720. The red points indicate significantly up‐regulated proteins. The blue points represent significantly down‐regulated proteins (*n* = 3; fold change > 2 and adj. Pval < 0.05); and the gray points represent proteins without significant differential changes. i) Expression abundance of proteins associated with platelet adhesion and immune escape properties in purified platelet membrane and PL720 (n = 3). j) Classification of PL720 proteins by molecular function. k) Classification of PL720 Proteins by biological process. Results are presented as mean ± SD.

**Figure** **2**a shows a schematic of the PL720 fabrication process using the PLT membrane extrusion method to load FTY720 and L‐arginine. FTY720 is an amphiphilic molecule that is inserted into the bilayer membrane during co‐extrusion and reassembly with the platelet membrane. L‐arginine is encapsulated in the inner water phase because of its water‐soluble properties.

The transmission electron microscopy (TEM) morphology of PL720 in Figure 2b shows that PL720 exhibits spherical structures with an average diameter of ≈200 nm, closely resembling the diameter of platelet membrane nanovesicles (PNVs). As shown in Figure 2c, compared to PLTs, the hydrodynamic diameter of PL720 was significantly reduced by ≈80%, similar to that of PNVs, indicating that the loading of FTY720 and L‐arginine had no significant effect on the size. The zeta (ζ) potential of PLTs, PNVs, and PL720 were −23.90 ± 2.36, −27.17 ± 1.24, and −31.43 ± 1.46 mV, respectively (Figure 2d). The change in hydrodynamic diameter of PL720 in saline over 0, 1, 2, 3, and 7 d was measured using dynamic light scattering (DLS) technique. The results in Figure 2e demonstrate that within 7 days, PL720 exhibited a consistently high level of colloidal stability with a low polydispersity index (PDI) below 0.2.

The loading efficiency of L‐arginine and FTY720 was determined to be 21.43±2.36% and 100% based on the standard curve (Figure S1, Supporting Information). The difference in the loading mechanism leads to a difference in the release rates of FTY720 and L‐arginine from PL720, which is the precise therapeutic effect of PL720 in different phases of MI/R. As shown in Figure 2f, L‐arginine exhibited a release rate of up to 60% within 1 h and 96% after 4 h. The rapid release of L‐arginine can effectively induce endothelial nitric oxide synthase‐mediated NO production and vasodilation, resulting in the rapid restoration of blood supply to ischemic regions in the early phase of MI/R. In contrast, FTY720 demonstrated a less than 20% release within 4 h and reached 20% release after 8 h (Figure 2f). The sustained release of FTY720 from PL720 can prevent rapid high‐dose FTY720 from significantly reducing the heart rate, thereby alleviating the resulting cardiac arrhythmias.

To characterize the retention of platelet membrane proteins in PL720 for its vascular injury‐targeting function, label‐free quantitative techniques were used to further investigate the protein composition of PL720. As shown in Figure 2g, after repeated freeze‐thaw cycles, the extracted platelet membrane retained 1500 proteins, which accounted for 84.41% (1500/1777) of the total protein species found in the original PLT population. PL720 retained 92.92% (1812/1950) of the total proteins in the extracted platelet membranes. Analysis of the differentially expressed proteins between the extracted platelet membrane and PL720 revealed that 55 proteins were up‐regulated and 94 were down‐regulated (Figure 2h). Most differentially expressed proteins were unrelated to cell interactions, platelet adhesion, and immune escape properties. Figure 2i shows the abundance of proteins associated with platelet adhesion and interactions with the immune system. Specifically, membrane integrin receptors and cell‐interaction proteins, such as GP1BA, GP1BB, GP5, GP6, GP9, CD62P, CD47, and CD58, have been identified in PL720, which is beneficial for its strong binding affinity to damaged blood vessels.^[^ ^14^ ^]^ To further characterize the binding ability of PL720 to the exposed damaged blood vessels, intact and denuded aortic arch vessels were incubated with DiD‐labeled PL720 for 1 min, followed by phosphate buffer saline (PBS) washing. Results show that PL720 exhibited better adherence to denuded vessels (Figure S2, Supporting Information), demonstrating the binding capability of PL720.

Proteins exhibiting no discernible differential expression between the purified platelet membrane and PL720 were categorized based on their molecular functions (Figure 2j) and biological processes (Figure 2k). These results demonstrate that PL720 inherits the abilities of natural PLTs.

### 2.2 In Vitro NO Production of PL720 in Endothelial Cells and Anti‐Apoptosis in Cardiomyocytes

**Figure 3:**
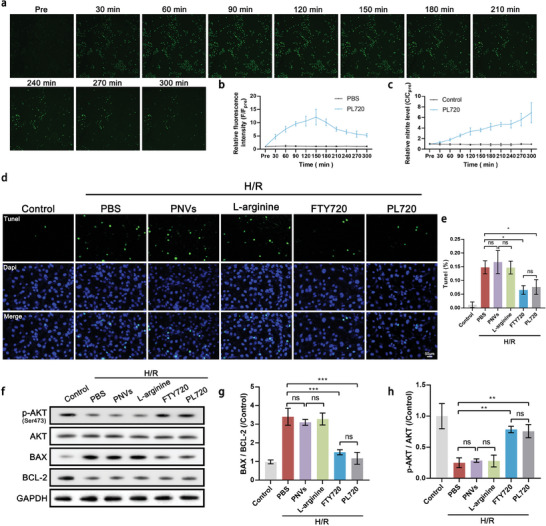
In vitro assessment of the anti‐apoptotic and NO‐generating capacity of PL720. a) CLSM images of HUVECs stained with DAF‐FM DA after interacting with PL720 at predetermined time points. b) Quantitative analysis of the mean fluorescence intensity of HUVECs in (a) (*n* = 3). c) Relative nitrite levels in the culture supernatants of HUVECs using the Griess reaction (*n* = 3). d) Evaluation of the anti‐apoptotic effects of PBS, PNVs, FTY720, L‐arginine, and PL720 on H/R‐treated H9C2 cardiomyocytes. e) Quantitative analysis of apoptosis percentage of H9C2 cells in (d) (*n* = 3). f) WB analysis of p‐AKT, AKT, BAX, and BCL‐2 protein levels. g) Quantitative analysis of p‐AKT/AKT levels in (f) (*n* = 3). h) Quantitative analysis of BAX/BCL‐2 levels (*n* = 3). Results are reported as mean ± SD. Data were analyzed using one‐way ANOVA followed by a two‐tailed Student's *t*‐test. ns indicates non‐significant (*p* > 0.05). ^*^ *p* < 0.05, ^**^ *p* < 0.01, and ^***^ *p* < 0.001.

Our previous results have demonstrated that adequate concentrations of NO at the site of injury can facilitate vasodilation, diminish thrombus formation, and enhance drug delivery.^[^ ^13^ ^]^ Thus, the in vitro production of NO by PL720 in endothelial cells was investigated by incubating PL720 with human umbilical vein endothelial cells (HUVECs), for 20 min. The NO‐sensitive fluorescent probe diaminofluorescein‐FM diacetate (DAF‐FM DA) was used to assess NO production. Intracellular NO production was determined by measuring the intensity of bright green fluorescence in the fluorescence images (**Figure** **3**a). Figure 3b shows that a bright green fluorescence enhancement is observed after 30 min when incubated with PL720 (200 µL), reaching its peak at 150 min and subsequently declining gradually. The nitrite level in the supernatant of the PL720‐exposed endothelial cell culture was quantitatively measured using the Griess reaction. Figure 3c shows the normalized relative nitrite levels. With the time increase of interaction between PL720 and HUVECs, the nitrite levels in the medium continued to increase. The absolute nitrite level was 0.92 µM after incubation for 300 min. There was an appropriately six‐fold increase compared to that before the addition of PL720, indicating that NO was sustainably released from HUVECs.

It has been reported that FTY720 activates S1PR1, leading to AKT phosphorylation and subsequently alleviating myocardial cell apoptosis.^[^ ^15^ ^]^ To evaluate the cardiomyocyte protective capability of PL720, an in vitro H9C2 cardiomyocyte hypoxic reoxygenation (H/R) model was prepared. After incubation with different samples (PNVs, L‐arginine, FTY720, and PL720) at the onset of reoxygenation for 12 h, anti‐apoptotic effects were investigated. Terminal deoxynucleotidyl transferase dUTP Nick End Labeling (TUNEL) staining results in Figure 3d,e demonstrates that PL720 exhibited a similar anti‐apoptotic effect with FTY720, resulting in the reduced apoptosis rate of cardiomyocytes to only 7.6 ± 2.7%. As a control, PNVs showed no anti‐apoptotic capability. Western blot (WB) analysis of B‐cell lymphoma 2 (BCL‐2) and Bcl‐2‐associated X protein (BAX) proteins showed that the BAX/BCL‐2 ratio underwent a 2.5‐fold downregulation for PL720‐treated H/R cardiomyocytes (Figure 3f,g). This alteration signified a substantial decrease in cardiomyocyte apoptosis. To investigate the potential association between the anti‐apoptotic activity of PL720 and the promotion of AKT phosphorylation, immunoblotting was conducted to assess the expression levels of AKT and phosphorylated AKT (p‐AKT) in cardiomyocytes (Figure 3f,h). WB results indicated that PL720 enhanced the expression of p‐AKT, thereby down‐regulating the BAX/BCL‐2 ratio and exerting an anti‐apoptotic effect. Overall, these findings suggest that PL720 has the potential to enhance NO production in endothelial cells and alleviate myocardial cell apoptosis following H/R.

The in vitro experiments using the Cell Counting Kit‐8 (CCK‐8) assay demonstrated that PL720 did not exhibit significant cytotoxicity against HUVECs or H9C2 cells (Figure S3, Supporting Information).

### 2.3 In Vitro Monocyte Binding and Macrophage Uptake

**Figure 4:**
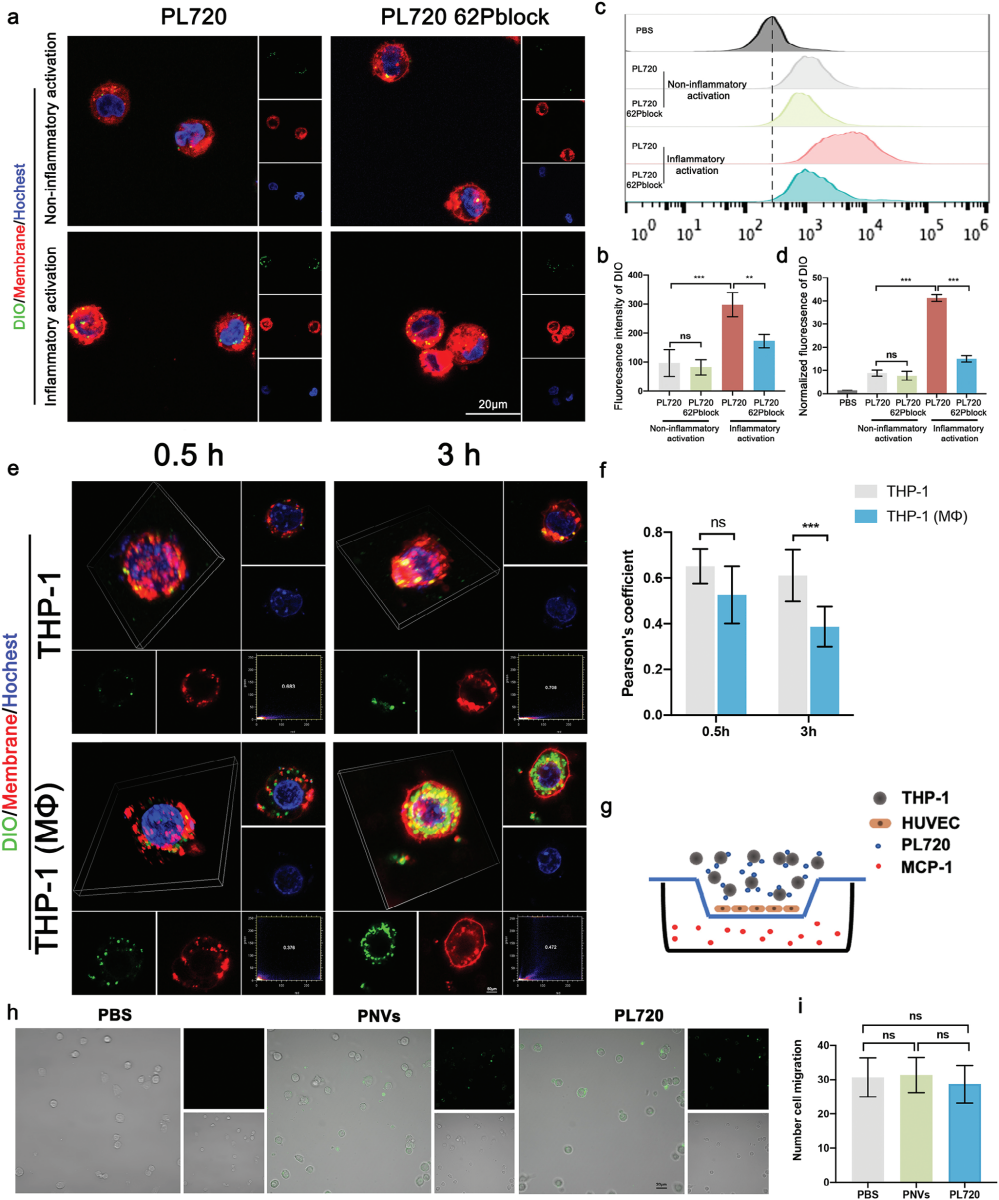
In vitro monocyte‐binding ability and macrophage uptake ability of PL720. a) Representative CLSM images of THP‐1 monocytes bound with DiO‐labeled PL720 and anti‐CD62P antibody blocked PL720 after pre‐incubation with or without LPS+INF‐γ stimulation (red: cell membrane, green: PL720, blue: nuclei). b) Semi‐quantification of DiO fluorescence intensity in (a) (*n* = 4). c) Flow cytometry analysis of THP‐1 monocytes bound with DiO‐labeled PL720 and PL720 62Pblock after pre‐incubation with or without inflammatory activation. d) Quantification of DiO normalized fluorescence intensity in (c) (*n* = 3). e) Representative CLSM images of monocytes (THP‐1) and macrophages (THP‐1 (MΦ)) after incubation with DiO‐labeled PL720 for 0.5 and 3 h. (Green: PL720, Red: cell membrane, blue: nuclei). f) The Pearson's correlation coefficient of membrane and PL720 was measured at different time points (*n* = 8). g) Schematic diagram of Transwell assay for endothelial penetration capacity of monocytes. h) Representative images of monocytes in the lower chamber after migration through the endothelial layer (green: PL720). i) Quantitative analysis of monocytes in the lower chamber after migration across endothelial layer (*n* = 3). Results are reported as mean ± SD. Data were analyzed using one‐way ANOVA followed by a two‐tailed Student's *t*‐test. ns indicates non‐significant (*p* > 0.05). ^*^ *p* < 0.05, ^**^ *p* < 0.01, and ^***^ *p* < 0.001.

*P*‐selectin glycoprotein ligand‐1 (PSGL‐1) exhibits robust expression in circulating Ly6C^+^ monocytes^[^ ^12^ ^a,^ ^16^ ^]^ and the binding of PSGL‐1 to *P*‐selectin (CD62P) on PLTs leads to aggregate formation. This mechanism facilitates the recruitment of both PLTs and monocytes to compromised heart tissue. Ly6C^+^ monocytes serve as the predominant source of inflammatory macrophages within the lesion region.^[^ ^17^ ^]^ To explore the potential targeted delivery of PL720 facilitated by monocytes, the binding ability of PL720 to monocytes and the formation of aggregates were investigated. **Figure** **4**a,b shows that compared to THP‐1 monocytes without inflammatory activation, inflammatory activated THP‐1 monocytes exhibited increased binding capability to PL720, with a two‐fold higher fluorescence intensity. This phenomenon is attributed to the upregulation of adhesion proteins and the modification of their properties in monocytes in response to inflammation.^[^ ^18^ ^]^ In the presence of non‐inflammatory activation, blocking CD‐62P had no significant effect on the aggregation of PL720 and THP‐1 monocytes. However, in the presence of inflammatory activation, blocking CD‐62P significantly reduced the aggregation of PL720 and THP‐1 monocytes (Figure 4a,b). Flow cytometry analysis further confirmed that after blocking CD62P in the presence of inflammatory activation, the binding efficiency of PL720 to THP‐1 monocytes was ≈40% of that observed in normal cells (Figure 4c,d). Therefore, PL720 inherited the ability of platelet membranes to adhere to inflammatory activated monocytes.

To understand the different interaction effects of PL720 on both monocytes and macrophages, THP‐1 cells and THP‐1 (MΦ) macrophages were subjected to inflammatory activation and incubated with 3, 3′‐dioctadecyloxacarbocyanine perchlorate (DiO)‐labeled PL720 for 0.5 and 3 h, respectively. The cell membrane was stained with red 1,1′‐dioctadecyl‐3,3,3′,3′‐tetramethylindocarbocyanine perchlorate (DiI) dye. Co‐localization of DiO and DiI cell membrane fluorescence was observed at 0.5 and 3 h in THP‐1 cells, with Pearson coefficients exceeding 0.5, suggesting the predominant adherence of PL720 to the cell membrane surface (Figure 4e,f). Compared with monocytes, PL720 exhibited co‐localization with the cell membrane of THP‐1 (MΦ) at 0.5 h. However, after 3 h, the green fluorescence appeared in the cytoplasm of THP‐1 (MΦ), suggesting that PL720 can be uptaken by inflammatory activation macrophages.

To confirm the potential impact of monocytes‐PL720 aggregates on monocyte inflammatory chemotaxis, Transwell experiments (Figure 4g) were conducted by seeding HUVECs in the upper chambers. Monolayer formation was confirmed when the transendothelial electrical resistance (TEER) stabilized above 25 Ω cm^2^. Following a 30 min pre‐treatment with PBS, PNVs, or PL720, inflammation‐activated THP‐1 cells were introduced into the upper chamber, while Monocyte chemoattractant protein‐1 (MCP‐1) were added to the lower chamber to facilitate monocyte chemotaxis. Figure 4h,i shows that the number of monocytes migrating to the lower chamber was ≈25–30 cells in all three groups, indicating that monocyte‐PL70 aggregates exhibited a similar endothelial penetration capacity as monocytes. Moreover, the CCK‐8 assay demonstrated that PL720 did not exhibit significant cytotoxicity in THP‐1 cells (Figure S3, Supporting Information).

### 2.4 In Vitro Immunomodulation Effect of PL720

**Figure 5:**
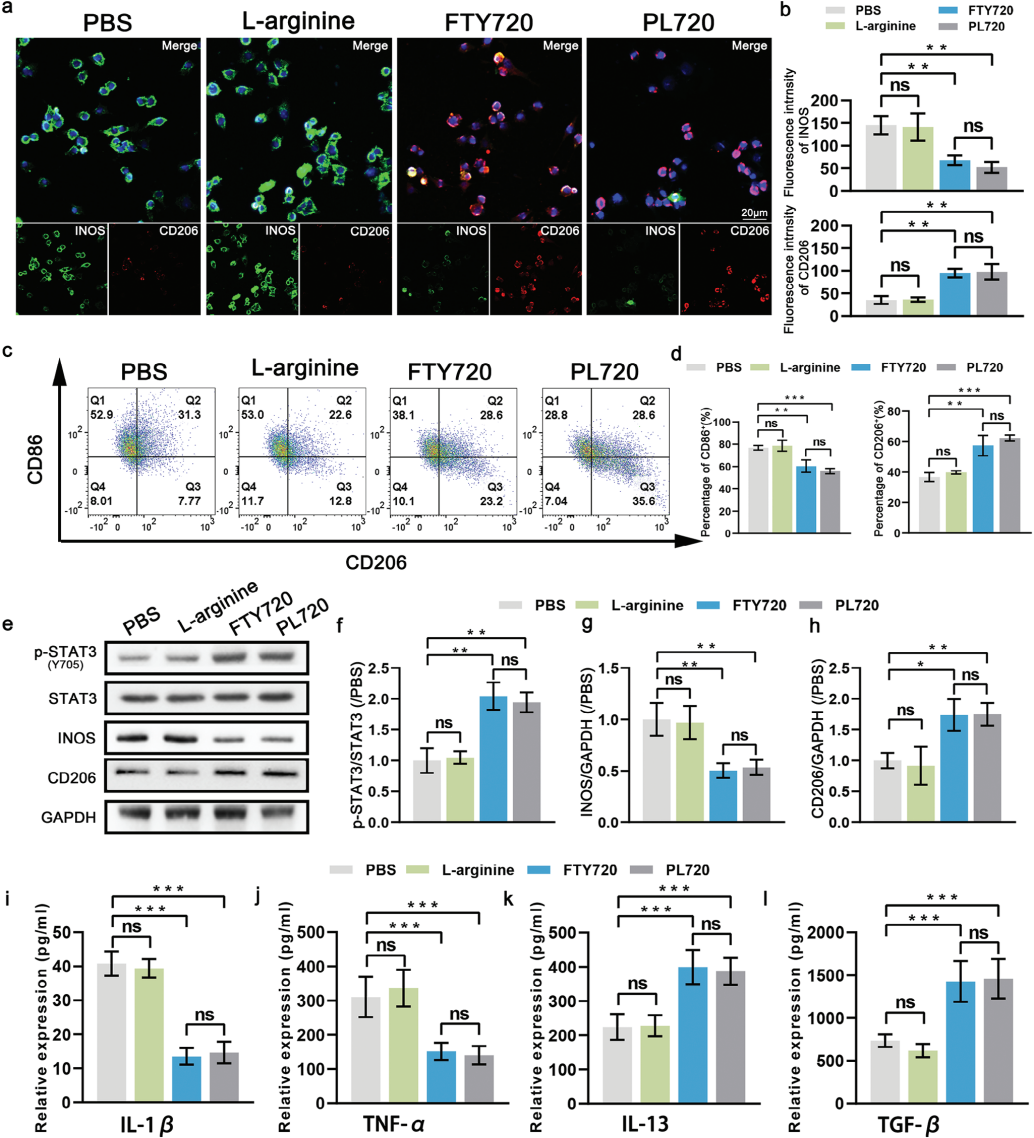
PL720 activates STAT3 pathway to induce immunomodulation in vitro. a) CLSM images indicate phenotypes of inflammatory BMDMs after being treated by PBS, FTY720, and PL720. b) Quantitative analysis of fluorescence intensity of iNOS (M1) and CD206 (M2) in (a) (*n* = 3). c) Flow cytometry assay of inflammatory BMDMs after treated by PBS, FTY720, and PL720. d) Statistical analysis of flow cytometry results in (c) (*n* = 3). e) WB of p‐STAT3, STAT3, INOS, and CD206 protein. f–h). Quantification of p‐STAT3/STAT3, INOS/GAPDH and CD206/GAPDH levels (*n* = 3). i–k) Concentration of cytokine M1 markers (IL‐1β, TNF‐α) and M2 markers (IL‐10, TGF‐β) in supernatants (*n* = 3). Results are reported as mean ± SD. Data were analyzed using one‐way ANOVA followed by a two‐tailed Student's *t*‐test. ns indicates non‐significant (*p* > 0.05). ^*^ *p* < 0.05, ^**^ *p* < 0.01, and ^***^ *p* < 0.001.

To examine the potential immunomodulatory effect of PL720, inflammatory bone‐marrow‐derived macrophages (BMDMs) (M1 macrophages) were treated with L‐arginine, FTY720, and PL720 for 24 h. The confocal laser scanning microscopy (CLSM) images in **Figure** **5**a reveals that, compared to the PBS treatment group, the fluorescence intensity of inducible nitric oxide synthase (iNOS; M1 phenotype marker) decreased by 53% in the FTY720 group and 60% in the PL720 group. No significant differences were observed between the PL720 and FTY720 groups (Figure 5b). In contrast, the fluorescence intensity of CD206 (an M2 phenotype marker) was increased by 168% in the FTY720 group and 177% in the PL720 group, respectively. In comparison with the PBS group, L‐arginine did not induce significant alterations in the fluorescence intensity of iNOS and CD206 (Figure 5b), suggesting no effect on the immune regulatory function. The flow cytometry results in Figure 5c,d are consistent with the fluorescence imaging results. After 24 h of treatment, the proportion of CD86^+^ (M1 phenotype marker) cells decreased, whereas the proportion of CD206^+^ cells increased in both the FTY720 and PL720 groups. No significant differences were observed between the PL720 and FTY720 groups. Both PL720 and FTY720 effectively triggered the conversion of M1 macrophages to M2 macrophages in vitro. Encapsulation of FTY720 within platelet membranes did not impair its immunomodulatory function.

To further investigate the mechanism, a protein blotting experiment was used to verify whether PL720 regulates the M1/M2 polarization of macrophages by activating STAT3 because the activation of STAT3 by FTY720 has been reported to promote the polarization of the macrophage M1 /M2 phenotype.^[^ ^8^ ^a,^ ^19^ ^]^ Similar to the FTY720 group, as shown in Figure 5e,f, the expression of phosphorylated STAT3 (p‐STAT3) in BMDMs was significantly upregulated by approximately two‐fold after 24 h of PL720 treatment. These findings demonstrate that PL720 exerts immunomodulatory effects by activating the STAT3 signaling pathway. Furthermore, WB analysis in Figure 5g,h also demonstrated down‐regulation of iNOS expression and increased expression of CD206 in BMDMs, providing further evidence that PL720 can modulate macrophage polarization.

In addition, the supernatant of the cell culture medium was collected to detect inflammatory gene expression using Enzyme linked immunosorbent assay (ELISA) test. Compared to the PBS group, results in Figure 5i–l demonstrated that both PL720 and FTY720 suppressed the production of Interleukin‐1 beta (IL‐1β) and TNF‐α (TNF‐α). Due to macrophage polarization, the production of Interleukin‐1 beta (IL‐13) and Transforming Growth Factor‐beta (TGF‐β) was enhanced. Moreover, the CCK‐8 assay demonstrated that PL720 did not exhibit significant cytotoxicity against BMDMs (Figure S3, Supporting Information).

### 2.5 In Vivo Biodistribution, Targeting, and Cardiac Treatment Effects with One Dose of PL720 Administration During Ischemic–Reperfusion Phase

**Figure 6:**
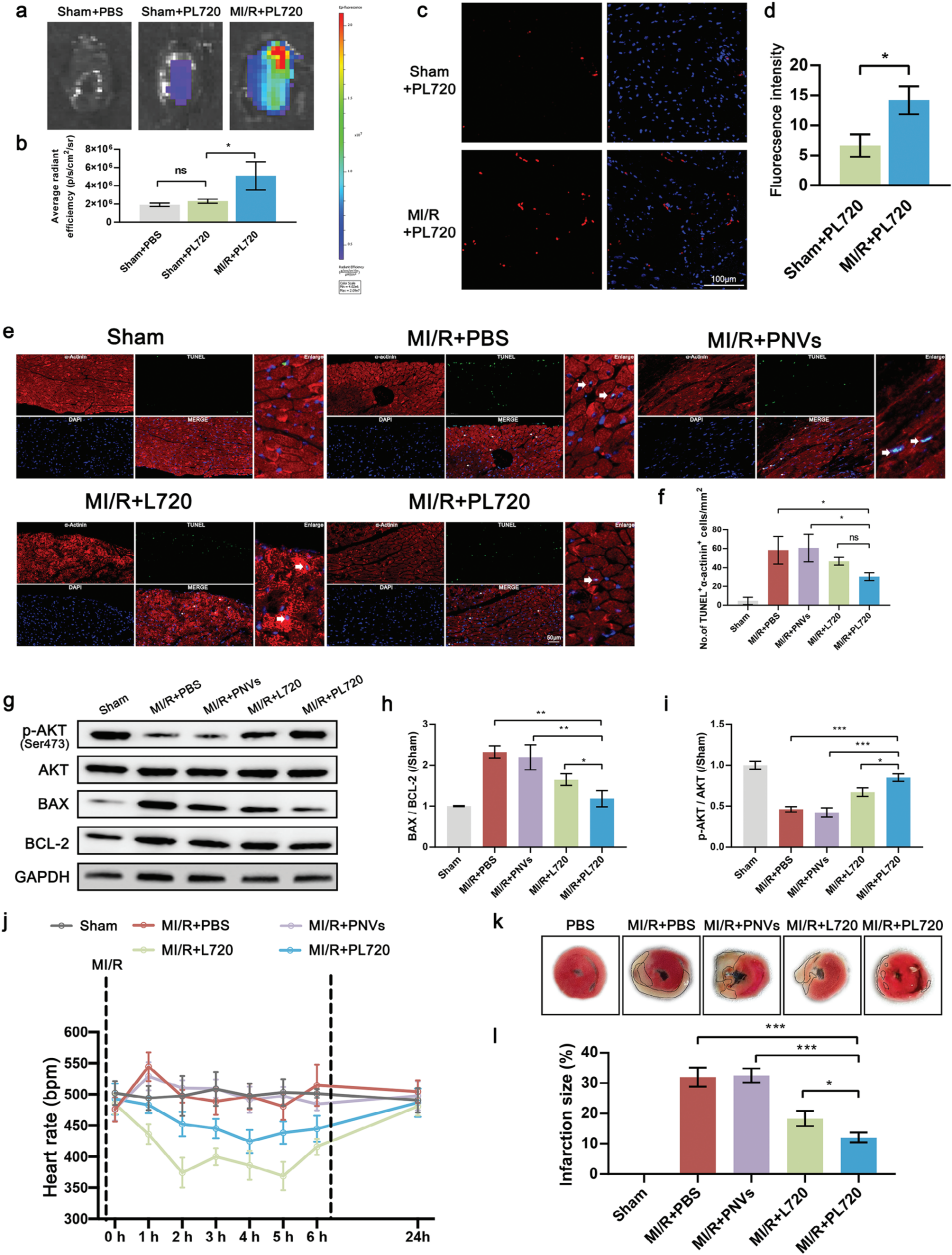
Targeting specificity, cardiomyocyte anti‐apoptotic effects, and the ability to rescue damaged myocardium after treatment with a single dose of PL720 during the ischemic phase. a) The typical ex vivo NIR fluorescence images of hearts from sham, sham+PL720, and MI/R + PL720 groups post‐injection for 6 h. b) The mean fluorescence intensity was obtained from the heart regions of mice described in (a) (*n* = 3). c) The CLSM image of the heart lesion 12 h after injection of DiI‐labeled PL720 for healthy (sham) and MI/R mice. d) Quantitative analysis of DiI fluorescence signal intensities in hearts in (c) (*n* = 3). e) Representative images of TUNEL staining 2d after surgery to detect apoptotic nucleus (white arrows point to apoptotic cardiomyocytes). f) The number of TUNEL positive staining nucleus in (e) (*n* = 3). g) WB of p‐AKT, AKT, BAX, and BCL‐2 protein. h) Quantification of p‐AKT/AKT levels (*n* = 3). i) Quantification of BAX/BCL‐2 levels (*n* = 3). j) Heart rates of mice in each group at different times (0, 1, 2, 3, 4, 5, 6, and 24 h) after the first injection of PBS, PNVs, L720, and PL720 (*n* = 6). k) TTC staining images of mouse heart in each group to evaluate myocardial ischemia injury. l) TTC staining was quantified to determine infarct size (*n* = 4). Results are reported as mean ± SD. Data were analyzed using one‐way ANOVA followed by a two‐tailed Student's *t*‐test. ns indicates non‐significant (*p* > 0.05). ^*^ *p* < 0.05, ^**^ *p* < 0.01, and ^***^ *p* < 0.001.

To visualize the cardiac targeting of PL720 during the ischemica reperfusion phase of MI/R, 1, 1′‐dioctadecyl‐3, 3, 3′, 3′‐tetramethylindotricarbocyanine iodide (DiR)‐labeled PL720 was injected into the tail veins of healthy (sham) and MI/R mice (Figure S4a, Supporting Information). After PL720 injection for 6 h after this phase, the mice were sacrificed and the major organs (hearts, brains, lungs, livers, spleens, and kidneys) were used for ex vivo fluorescence imaging to investigate the biodistribution of PL720. The results in **Figure** **6**a,b demonstrates that the cardiac targeting efficiency of PL720 in the PL720‐treated MI/R group was significantly enhanced. Compared to the PL720‐treated sham group, it had 2.20 times higher accumulation efficacy. In contrast, the MI/R+PL720 group exhibited reduced accumulation of PL720 in the liver and spleen compared with the sham+PL720 group. The distribution in the other major organs did not show any significant differences (Figure S5, Supporting Information). To further understand the PL720 targeting to damaged coronary arteries and recognition of ischemic myocardial lesions in MI/R mice, DiI‐labeled PL720 was administrated via tail vein injection to both sham and MI/R mice. Post 12 h of injection, the hearts of the mice were extracted and frozen for observation using a fluorescence microscope. As shown in Figure 6c,d, the fluorescence intensity in the MI/R+PL720 group was notably higher than that in the Sham+PL720 group, confirming that PL720 can target the heart and enter deeper ischemic myocardial lesions.

Furthermore, the TUNEL staining was performed to assess cardiomyocyte apoptosis in the infarcted region of each mouse group. In contrast to the in vitro cardiomyocyte apoptosis results, the in vivo results in Figure 6e,f demonstrates a stronger anti‐apoptotic effect of PL720 than that of L720. This can be attributed to the targeting ability and sustained‐release effect of PL720 in ischemic reperfusion myocardial lesions. In addition, the expression of AKT, p‐AKT, BAX, and BCL‐2 in the hearts of each mouse group is shown in Figure 6g–i suggesting that the PL720‐treated mice exhibited the highest expression of p‐AKT and the lowest BAX/BCL‐2 ratio among all treatment MI/R groups.

Although the anti‐apoptotic and anti‐inflammatory effects of FTY720 have been documented,^[^ ^20^ ^]^ the initial standard treatment dose triggers bradycardia owning to the rapid activation of S1P receptors, which considerably limits its applicability in heart disease treatment, especially in the context of ischemic cardiomyopathy.^[^ ^21^ ^]^ To assess the impact of the sustained, low‐dose release of FTY720 from PL720, the heart rate of mice at pre‐determined time points (1, 2, 3, 4, 5, 6, and 24 h) after PL720 administration were monitored. Figure 6j shows that after PL720 treatment for 4 h, the mouse heart rate decreased to a nadir of 425 beats per minute (bpm), sustaining at ≈450 bpm within 6 h period. However, in the L720 treatment group, after 2 h, the mouse heart rate decreased to a nadir of 374 bpm, was continuously maintained below 400 bpm for 5 h, and increased to 415 bpm at 6 h. After 24 h, the heart rates of all mice returned to baseline levels. Furthermore, as shown in Figure S6 (Supporting Information), after the second dose administration of PL720 at the inflammatory phase, the heart rate is stable. However, for L720 treated mice, the heart rate decreased to 450 bpm at the initial 6 h and then returned to baseline levels. Notably, the administration of PNVs did not affect the heart rate of the mice. These findings suggest that sustained low‐dose release of FTY720 from PL720 is beneficial for mitigating the impact of FTY720 on heart rate, thereby enhancing its safety.

The in vitro and in vivo experiments demonstrated the ability of PL720 to inhibit cardiomyocyte apoptosis and enhance NO production. Triphenyltetrazolium chloride (TTC) staining was used to characterize the therapeutic effect and assess the infarct size (Figure 6k). TTC staining analysis in Figure 6l reveals that mice treated with PL720 exhibited a smaller cardiac infarct size than those treated with L720. The PNVs had no impact on infarct size.

### 2.6 In Vivo Biodistribution, Targeting, and Immune Regulation Effects with One Dose of PL720 Treatment During Late Reperfusion Inflammatory Phase

**Figure 7:**
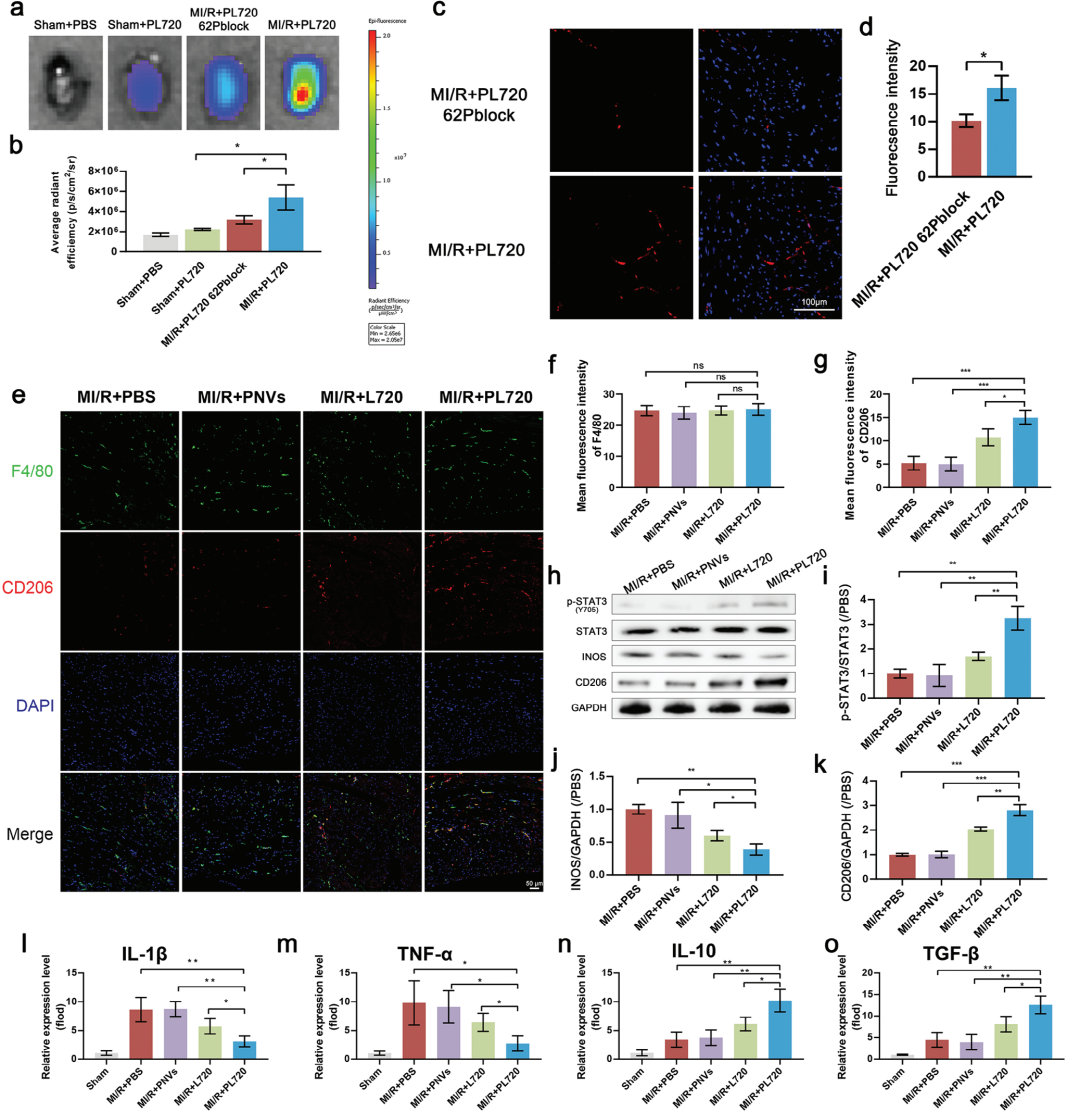
Targeting specificity, and macrophage polarization reprogramming effect of a single dose of PL720 during the late reperfusion inflammation phase. a) The typical ex vivo NIR images of the heart for sham group or MI/R mice injected with DiR‐labeled PL720 or PL720 62Pblock. b) Quantitative analysis of DiR fluorescence signal in hearts for different groups of mice (*n* = 3). c) The CLSM image of the injury site in the heart after injection of DiI‐labeled PL720 and PL720 62Pblock. d) Quantitative analysis of DiI fluorescence signal in hearts for different groups of mice (*n* = 3). e) CLSM images of MI/R injured heart sections showing the total (F4/80) and M2 subtype (CD206) macrophages after being treated by PBS, PNVs, L720, and PL720, respectively. f) Fluorescence intensity quantification of F4/80 in (e) (*n* = 4). g) Fluorescence intensity quantification of CD206 in (e) (*n* = 4). h) WB of p‐STAT3, STAT3, INOS, and CD206 protein levels. i–k) Quantification of p‐STAT3/STAT3, INOS/GAPDH and CD206/GAPDH levels (*n* = 3). l–o) Quantification of L‐1β, TNF‐α, IL‐10, and TGF‐β related mRNA expression (*n* = 3). Results are reported as mean ± SD. Data were analyzed using one‐way ANOVA followed by a two‐tailed Student's *t*‐test. ns indicates non‐significant (*p* > 0.05). ^*^ *p* < 0.05, ^**^ *p* < 0.01, and ^***^ *p* < 0.001.

To assess the targeting capacity of PL720 in the late reperfusion inflammatory phase of the heart after MI/R, DiR‐labeled PL720 or PL720 62Pblock was intravenously injected into MI/R and sham mice 72 h post‐surgery (Figure S4b, Supporting Information). At 6 h post‐PL720 injection, the mice were euthanized, and the hearts, brains, lungs, livers, spleens, and kidneys (Figure S7, Supporting Information) were collected for ex vivo fluorescence imaging to examine the in vivo distribution of PL720. **Figure** **7**a demonstrates the significantly enhanced cardiac‐targeting efficiency of PL720 in the MI/R+PL720 group, which was 2.42 times higher than that in the sham+PL720 group (Figure 7b). The targeting efficiency of the MI/R+ PL720 62Pblock group was only 58.74% compared to that of the MI/R+PL720 group. These findings were consistent with the cellular results, indicating that after blocking CD62P on the membrane surface of PL720, its ability to bind to monocytes was weakened, leading to a diminished capacity to target the heart. Compared to the MI/R+PL720 group, the other groups showed more PL720 retention in the liver and spleen.

To further examine the ability of PL720 to enter myocardial tissue in the context of the cardiac inflammation phase. After a 12 h interval, cryo‐sections of the lesion area were examined using a fluorescence microscope (Figure 7c). The results showed that the fluorescence intensity of the MI/R+PL720 group was significantly higher than that of the MI/R+ PL720 62Pblock group (Figure 7d), which further indicated that the cardiac enrichment ability of PL720 was also weakened when CD62P was blocked.

Furthermore, the inflammatory phenotype in the lesion region of the heart was investigated by evaluating the effect of PL720 on the macrophage phenotype using immunofluorescence staining. As shown in Figure 7e, compared with the L720 treatment group, the total number of macrophages (F4/80+ cells) showed no significant change after PL720 treatment; however, there was a significant increase in the proportion of M2 subtype macrophages (CD206+ cells) (Figure 7f,g). The WB analysis further corroborated these findings, revealing that PL720 treatment led to the lowest expression of iNOS and the highest expression of CD206 in the injured tissue area (Figure 7h–k). The WB results in Figure 7h,i demonstrates a significant increase in phosphorylated STAT3 after PL720 treatment, indicating activation of the STAT3 signaling pathway to facilitate the polarization ratio of M1/ M2. The real‐time PCR (RT‐PCR) analysis was performed to assess the concentration of inflammatory cytokines (IL‐1β, TNF‐α, TGF‐β, and IL‐10) in the heart lesion of MI/R‐induced mice after treatment with PL720. The results revealed a significant reduction in IL‐1β and TNF‐α concentrations (Figure 7l,m), and a notable increase in TGF‐β and IL‐10 concentrations (Figure 7n,o) after PL720 treatment. Taken together, these findings provide compelling evidence that, during the late reperfusion inflammation phase in MI/R injury, PL720 effectively targets inflammatory regions in the heart through monocytes. By activating the STAT3 signaling pathway, PL720 facilitates the reparative polarization of inflammatory macrophages.

### 2.7 Protective Effect of Two Doses of PL720 Administration on Cardiac Function

**Figure 8:**
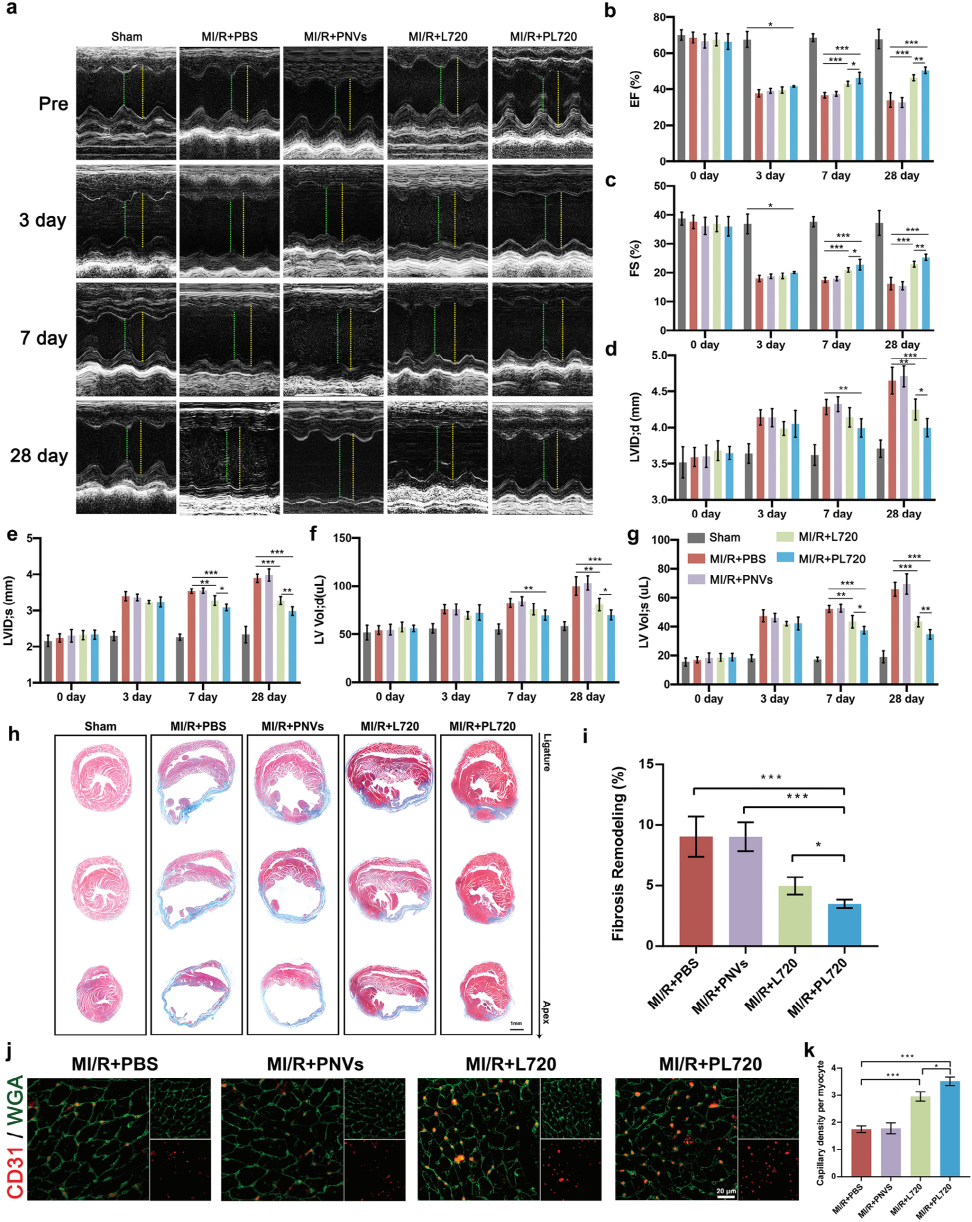
Cardioprotection effect after two doses of PL720 treatment. a) Echocardiograms obtained by M‐mode ultrasound at different times post‐operation (pre, day 3, day 7, and day 28). b–g) Cardiac function of the mice was evaluated according to the EF, FS, LVID; d, LV Vol; d, LVID; s and LV Vol; s measured by the echocardiography of the mice in each group (*n* = 5). h) Masson staining of MI/R heart paraffin sections. i)Fibrosis remodeling percentage of MI/R mice after treatment with PBS, PNVs, L720, and PL720 based on Masson staining images. j) Sections of the injury sites in each group of mice were subjected to CD31 and WGA staining. CD31 positivity was utilized to visualize microvessel at the boundary areas of the injury sites, while WGA staining was utilized to visualize myocyte in the border regions of the injury area. k) Quantification of the number of CD31 positive and myocyte within the equivalent area of the injury border region, and computation of their ratio (*n* = 3). Results are reported as mean ± SD. Data were analyzed using one‐way ANOVA followed by a two‐tailed Student's *t*‐test. ns indicates non‐significant (*p* > 0.05). ^*^ *p* < 0.05, ^**^ *p* < 0.01, and ^***^ *p* < 0.001.

To evaluate the overall protective effect of PL720 on MI/R hearts, different samples (PBS, PNVs, L720, and PL720) were administered to MI/R mice via the tail vein at two target time points (ischemia/reperfusion phase and late reperfusion inflammation phase) (Figure S4c, Supporting information). Initially, echocardiographic assessments were conducted to evaluate cardiac function in MI/R‐induced mice before surgery and at 3, 7, and 28 d after surgery (**Figure** **8**a). Parameters such as left ventricular ejection fraction (EF), fractional shortening (FS), left ventricular internal diameter at end‐diastole (LVID; d), left ventricular internal diameter at end‐systole (LVID; s), left ventricular end‐diastolic volume (LV Vol; d), and left ventricular end‐systolic volume (LV Vol; s) did not exhibit significant differences among the groups on day 3 (Figure 8b‐g). Compared to the PBS treatment group, both the L720 and PL720 treatment groups showed significant increases in EF and FS on day 7. There was a 3.21% increase in EF and a 1.81% increase in FS in the PL720 treatment group compared to the L720 treatment group (Figure 8b,c). Moreover, on the day 7, both the L720 and PL720 treatment groups exhibited significant reductions in LVID; s (3.54 mm vs 3.27 mm vs 3.08 mm, MI/R+PBS vs MI/R+L720 vs MI/R+PL720) and LV Vol; s (52.17 µL vs 43.40 µL vs 37.46 µL, MI/R+PBS vs MI/R+L720 vs MI/R+PL720) (Figure 8e,g). The PL720 treatment group showed a greater reduction than the L720 treatment group. It is noteworthy that only the PL720 treatment group demonstrated a significant decrease in LVID; d (4.287 mm vs 3.993 mm, MI/R+PBS vs MI/R+PL720) and LV Vol; d (82.512 uL vs 69.805 uL, MI/R+PBS vs MI/R+PL720) compared to the PBS treatment group at day 7 (Figure 8d,f). After 28 days, the PL720 group exhibited the highest increase in EF (51.19%) and FS (26.05%) (Figure 8b,c), as well as the lowest increase in LVID; d (3.40 mm), LVID; s (2.99 mm), LV Vol; d (69.99 µL), and LV Vol; s (34.66 µL) (Figure 8d‐g). Compared to the L720 treatment group, the EF and FS of the PL720 treatment group increased by 5.16% and 2.99% (Figure 8b,c), respectively. The preservation efficiency of heart shape and size, as shown in Figure 8d–g, was prominent in the PL720 treatment group.

Moreover, cardiac remodeling was assessed by Masson staining of different layers of cardiac paraffin sections and by quantifying the overall extent of fibrosis. Consistent with the results obtained from TTC staining and echocardiography, the hearts of mice that received two doses of PL720 exhibited the highest preservation of the viable myocardium and the smallest fibrotic area (9.04% vs 4.96% vs 3.48%, MI/R+PBS vs MI/R+L720 vs MI/R+PL720) compared to the other groups (Figure 8h,i). Previous studies demonstrated that the M2 subtype contributes to the formation of sprouting vessels.^[^ ^22^ ^]^ The immunohistochemical analysis using CD31 markers was performed to characterize vessel formation. The PL720 treatment group exhibited the highest microvessel density (Figure 8j,k). These findings indicate that PL720 possesses vasodilator and anti‐apoptotic properties during the acute phase after MI/R. The regulation of macrophage polarization during the inflammatory phase promotes the maturation of new blood vessels in the heart.

### 2.8 Biosafety Assessment of PL720

Finally, the biosafety of two doses of PL720 was evaluated after treatment with PBS, PNVs, L720, or PL720. Liver and kidney functions were assessed in each group to identify any signs of acute liver or kidney injury on the 6th day after surgery. Results in Figure S8 (Supporting Information) show that the expression level of alanine aminotransferase (ALT), aspartate aminotransferase (AST), γ‐Glutamyltransferase (γ‐GT), urea nitrogen (UREA), and creatinine (CREA) were within the normal range. After 28 days of treatment, the Hematoxylin and Eosin staining results (Figure S9, Supporting Information) demonstrated that there were no histopathological changes in the major organs in the PL720 treated healthy mice group.

Furthermore, it is crucial to consider the effect of PL720 on coagulation function when employing a platelet membrane. Activated partial thromboplastin time (APTT), and prothrombin time (PT) were assessed on the 4th day after surgery. The results in Figure S10 (Supporting Information) reveal that PL720 does not exert any adverse effects on the coagulation and fibrinolytic systems. Collectively, these results demonstrated that PL720 exhibits excellent biological safety, which is essential for its potential clinical applications.

## 3 Conclusion

In this study, we developed a delivery platform utilizing platelet membrane‐encapsulated L‐arginine and FTY720 to target different phases of injured myocardial lesions in MI/R‐bearing mice. Such PL720 nanoplatforms enable sustained release of FTY720 and facilitate cascade targeting during the early phases of ischemic reperfusion and late reperfusion inflammation, thereby enabling a combined therapeutic effect with vasodilation, anti‐apoptosis, and immune modulation. This study had two major findings. First, by administering one dose of PL720 immediately after reperfusion, PL720 can reach the damaged coronary artery through recanalized blood flow. Upon reaching the injured site, PL720 promptly releases L‐arginine to facilitate NO production by endothelial cells. Simultaneously, sustained release of FTY720 inhibited cardiomyocyte apoptosis. Second, by administering one dose of PL720 three days after reperfusion, PL720 can bind to circulating Ly6C^+^ monocytes and be transported to the injured site because of the impaired cardiac chemotaxis of Ly6C^+^ monocytes, leading to anti‐inflammatory reprogramming of inflammatory macrophages. Therefore, PL720 shows cascade targeting capability for different phases of MI/R.

A notable advantage of PL720 is its alignment with the current concept of multiple ischemia–reperfusion therapy, which alleviates the adverse effects associated with FTY720. Although several studies have demonstrated the efficacy of FTY720 in the treatment of MI/R and AMI, its clinical application has yet to be realized. The primary obstacle lies in the initial use of FTY720, which affects heart rate and restricts its use for cardiovascular disease treatment. Our data shows that compared to FTY720, PL720 not only exhibits superior in vivo therapeutic effects but also has a lesser impact on heart rate, maintaining it within a relatively safe range following initial administration. Therefore, our study holds great promise for the clinical application of FTY720 in the cardiovascular field and offers translational potential for managing cardiomyopathy.

## 4 Experimental Section

### Materials

Apheresis platelets were obtained from the Blood Center of Jiangsu Province China. L‐arginine was obtained from Sigma–Aldrich (St. Louis, MO, USA). Fingolimod (FTY720) hydrochloride was purchased from Selleck (Houston, TX, USA). H9C2 cells, THP‐1 cells, and HUVECs were obtained from the Chinese Academy of Sciences Cell Bank (Shanghai, China). RPMI 1640 and Dulbecco's Modified Eagle's medium (DMEM) were obtained from KeyGEN Biotech (China). DiO (excitation/emission wavelength of 483/ 501, green color), DiR (excitation/emission wavelength of 754/ 778), and DiI (excitation/emission wavelength of 551/569, red color) were acquired from Beyotime (Haimen, China).

### Preparation of PL720 Nanocarriers

The fabrication process of PL720 involved a membrane‐extrusion method, consisting of the following steps based on previously developed method^[^ ^13^ ^]^: 1) The platelet membrane extraction and purification from human PLTs; 2) L720 solution preparation (i.e., a mixed solution of FTY720 and L‐arginine at concentrations of 0.1 mg mL^−1^ and 10 mm, respectively); 3) Subjected to 0.22 µm membrane filtration; 4) Resuspension of the purified platelet membrane with the prepared L720 solution; 5) Extrusion of the suspension through nuclepore polyester porous membranes to assemble as nanocarrier. The production process of PNVs was the same as that of PL720, but without the addition of L‐arginine and FTY720.

### Characterization of PL720 Nanocarriers

The microscopic morphology of PL720 was examined via TEM (JEM‐2100, JEOL, Japan) following negative staining with 1% phosphotungstic acid. The hydrodynamic size distribution and surface zeta potential (ξ) of PLTs, PNVs, and PL720 were determined using a Malvern NanoSizer (Zeta–Sizer, Malvern Instruments, UK). The 7‐day in vitro stability of PL720 was further evaluated by measuring its size change.

To confirm the composition and retention of the platelet membrane protein function of PL720, quantitative proteomic analysis of PLTs, purified platelet membranes, and PL720 was conducted using protein‐label‐free quantitative technology.

### Cell Culture

Three cell lines, namely HUVECs, rat cardiomyocytes H9C2, and monocyte‐like cell line THP‐1, along with primary BMDMs, were utilized in the study.

HUVECs and H9C2 cells were cultured in a DMEM complete medium. Cells were cultured, and upon reaching 70–80% confluence, detached using 0.25% trypsin‐EDTA solution. Subsequently, the cells were harvested by centrifugation and resuspended in PBS for experimental purposes.

THP‐1 cells and THP‐1‐differentiated macrophages (THP‐1(MΦ), THP‐1 treated with phorbol myristate (100 ng mL^−1^ for 3 days) were cultivated in RPMI 1640 complete medium. The cells were used for experiments when their density reached 1 × 10^5^.

BMDMs were isolated from 6 to 8‐week‐old C57BL/6 mice as previously described with minor modifications.^[^ ^23^ ^]^ Briefly, the mice were sterilized using 75% alcohol and sacrificed by rapid cervical dislocation. The tibia and femur were isolated. The bone marrow cavity was washed with sterile PBS, and centrifuged to remove the supernatant. The red blood cell lysate was added and centrifugation was performed to obtain a BMDM single‐cell suspension. The isolated cells were cultured in DMEM supplemented with 10% fetal bovine serum (FBS) and 40 ng mL^−1^ recombinant human macrophage colony‐stimulating factor (M‐CSF, Thermo Fisher Scientific, USA) for 7 days to promote the maturation of macrophages.

To prepare the inflammatory cell model, the THP‐1, THP‐1 (MΦ), and BMDM cells were stimulated with lipopolysaccharide (LPS, 100 ng mL^−1^) and human interferon‐γ (IFN‐γ, 20 ng mL^−1^) for a duration of 6 h to induce an inflammatory state of the cells, a phenomenon known as inflammatory activation.

### In Vitro NO Generation and Characterization

To quantify the production of NO when PL720 interaction with HUVECs, a NO fluorescent probe DAF‐FM DA (Beyotime, China) was added to HUVECs solution at a final concentration of 5 µm. After incubation in the dark for 20 min in a cell culture incubator, the unloaded probes were eliminated by washing the cells three times with PBS. Next, PL720 (20 µL) was added to each well of a six‐well plate. Real‐time NO generation was monitored using CLSM (Nikon C2, Japan) to capture fluorescence images at predetermined time intervals (every 30 min for 5 h). Fluorescence was quantified using Image J software (NIH, USA). Because of the potential influence of fluorescence on cell viability and the susceptibility of fluorescence to quenching, the nitrite level in the culture supernatant was measured using a Griess kit (Beyotime, China) to indirectly assess NO content. Absorbance was measured at 540 nm using a Multiskan SkyHigh microplate reader (Thermo Scientific, USA) following the manufacturer's instructions.

### In Vitro Apoptosis Assay

To determine H9C2 apoptosis efficiency when interacting with PL720, six groups were designed: normal H9C2 cells as the control group, and H/R H9C2 cells treated with PBS, PNVs, L‐arginine, FTY720, and PL720.

H9C2 cells were cultured in six‐well plates until they reached 70% confluence. The sugar‐free DMEM medium was then replaced, and the cells were incubated in a three‐gas incubator (1% O_2_, 5% CO_2_, 94% N_2_) at 37 °C for 6 h. Afterward, the oxygen supply was restored, and the cells were transferred to a high‐glucose DMEM medium containing 10% FBS. Subsequently, a total of 10 µL different samples (PBS, PNVs, L‐arginine, FTY720, and PL720) were added to the cells and incubated for another 12 h. Apoptotic rate determination was performed using the TUNEL kit (Beyotime, China) and 4′, 6‐Diamidino‐2′‐phenylindole (DAPI) cell nuclear staining (Beyotime, China) following the manufacturer's instructions. Finally, WB analysis was conducted.

### PL720 Nanocarriers Binding to Cell Experiment

To evaluate the binding capacity to monocytes, first, PL720 was incubated with an anti‐CD‐62P antibody (Santa Cruz, USA) for 8 h to inhibit CD‐62P interference, which is referred as PL720 62Pblock. Both inflammatory and non‐inflammatory activated THP‐1 cells were incubated with green DiO‐labeled PL720 or CD‐62P‐inhibited PL720 for 60 min. THP‐1 cells were stained using a DiI (red) cell membrane staining kit (Beyotime, China) and observed using CLSM. Quantitative analysis of cellular DiO fluorescence intensity post‐interaction with DiO‐labeled PL720 was performed using flow cytometry (Attune NxT, Thermo Fisher Scientific, USA).

### Macrophage Phagocytosis Assay

Both inflammatory activation THP‐1 cells and THP‐1(MΦ) were incubated with green DiO‐labeled PL720 for 30 min and 3 h, respectively. The cell membrane was stained using a DiI (red) cell membrane staining kit (Beyotime, China) to be observed under CLSM with an oil immersion lens (100×). The localization of PL720 in cells was analyzed using ImageJ software based on fluorescence signals.

### In Vitro Monocyte‐PL720 Binding

To investigate the chemotaxis ability of monocytes‐PL720 aggregate, HUVECs were inoculated into the upper chamber (Labselect, China) of a Transwell system with a pore size of 3 µm. The TEER of the upper endothelial cells was measured using Millicell ERS2 (Merck, USA), and a stable resistance value (>25 Ω cm^2^) was obtained through daily monitoring.^[^ ^24^ ^]^ Inflammatory activation was induced in both HUVECs and THP‐1 cells using the aforementioned procedures. THP‐1 cells were co‐incubated with DiO‐labeled PNVs and PL720 for 30 min, and then added to the upper chamber. MCP‐1 (100 ng mL^−1^; Beyotime, China) was added to the lower chamber. After incubation for 24 h, the medium from the lower chamber was collected and quantified using Countess II (Thermo, USA). In addition, the culture medium from the lower chamber was used to prepare cell slides, which were then observed under CLSM.

### In Vitro Macrophage Polarization State Assays

In the in vitro macrophage polarization experiment, BMDMs were cultured in 12‐well plates and induced into an inflammatory state. Then, they were randomly divided into four groups and treated with 10 µL PBS, L‐arginine, FTY720, and PL720, respectively for 24 h. Immunofluorescence staining was carried out using mouse anti‐iNOS antibody (eBioscience) and rabbit anti‐CD206 antibody (Abcam), followed by incubation with corresponding secondary antibodies (Beyotime, China). For flow cytometry analysis, PE‐Cy7‐anti‐ CD86 (eBioscience, USA) and APC‐anti‐CD206 (eBioscience, USA) were used for labeling. The levels of inflammatory factors, including IL‐1β (Mlbio, China), IL‐13 (Multi sciences, China), TNF‐α (Multi sciences, China), and TGF‐β (Multi sciences, China) were determined using ELISA kits following the manufacturer's instructions. The pro‐regenerative M2 phenotype was achieved by treating BMDMs with interleukin‐4 (IL‐4, PeproTech, USA) at a concentration of 20 ng mL^−1^ for 24 h. Finally, WB analysis was preformed.

### Animal Model Preparation, Experimental Protocol, and Histopathological Examination

Male C57BL/6 mice, aged 8–10 weeks, 22–25 g, were obtained from Nanjing Cavans Biotechnology Co., Ltd., China. All animal experiments were conducted in accordance with the Guidelines for the Care and Use of Laboratory Animals established by the Institutional Animal Care and Use Committee of the Medical School of Southeast University. A mouse model of myocardial ischemia–reperfusion was established based on a modification of a method described previously.^[^ ^25^ ^]^ In brief, mice were continuously anesthetized with 1–2% isoflurane. The pectoralis major and minor muscles were isolated and the pleura was punctured to extrude the heart from the chest cavity. The left anterior descending artery was ligated using a slipping knot. A small animal electrocardiograph (A‐01; Hinbon, China) was used to monitor the electrocardiogram (ECG). The mice were considered to have AMI when the ST (isoelectric section of the ECG between the end of the S wave (the J point) segment) exhibited an elevation resembling a dorsal arch (Figure S11, Supporting Information). After 1.25 h post‐ ischemia, the ligature was released, and the mouse MI/R model was established.

The mice that underwent thoracotomy without ligation were included in the sham group. The MI/R mice were randomly divided into four groups: Group 1, MI/R mouse tail vein injected with PBS (10 µL g^−1^); Group 2, MI/R mouse tail vein injected with PNVs (10 µL g^−1^); Group 3, MI/R mouse tail vein injected with L720 (10 µL g^−1^); Group 4, MI/R mouse tail vein injected with PL720 (10 µL g^−1^). The injection volume was based on the body weights of the mice.

To investigate the distinct therapeutic mechanisms of PL720 during the ischemia–reperfusion and late reperfusion inflammation phases and to elucidate the combined therapeutic effect of dual‐phase PL720, the three experimental protocols are shown in Figure S4 (Supporting Information).

Ischemia–Reperfusion phase treatment (Figure S4a, Supporting Information): After MI/R surgery, different samples (PBS, PNVs, L720, and PL720) were administered. The heart rates of the mice within 6 h and at the 24 h time point post‐surgery were monitored using an EGG. The mice were euthanized at 48 h postoperatively. Hearts were extracted from mice in each group (*n* = 4) and sectioned into 1 mm slices along the transverse axis. These slices were then immersed in a 1% solution of TTC (Sigma, USA) and incubated in a light‐free environment at 37°C for 15 min. The samples were fixed in 4% paraformaldehyde (Beyotime, China) and subjected to TTC staining to measure the extent of infarction. Furthermore, hearts from mice in each group (*n* = 4) were embedded in paraffin and sectioned with 10 µm slices. According to the manufacturer's instructions, the heart sections were subjected to staining using alpha‐smooth muscle actin (α‐actin, Sigma, USA), DAPI, and TUNEL (Beyotime, China). An inverted microscope (Olympus ckx53, Japan) was used to observe the extent of cardiomyocyte apoptosis in infarcted regions. Finally, WB analysis was conducted.

Late reperfusion inflammation phase treatment (Figure S4b, Supporting Information): At 72 h time point after MI/R surgery, different samples (PBS, PNVs, L720, and PL720) were administered. On the 6th day post‐surgery, the mice were euthanized, and hearts from each group (*n* = 4) were collected. The cardiac tissues were frozen and sectioned. Immunofluorescence staining was conducted using rabbit anti‐CD206 antibody (Abcam, USA), rat anti‐F4/80 antibody (Abcam, USA), and the corresponding secondary antibodies (Beyotime, China). Finally, WB and RT‐PCR were performed.

Two‐phase combined treatment (Figure S4c, Supporting Information): Immediately after MI/R surgery and at the 72 h time point, two doses of PBS, PNVs, L720, and PL720 were adminstrered. On the 8th day post‐surgery, mice from each group (*n* = 4) were euthanized, and the hearts were paraffin‐embedded and sectioned in 10 µm slices. Immunofluorescence was performed using a rabbit anti‐CD31 antibody (Sigma, USA) to evaluate neovascularization. Myocyte size was assessed by Wheat Germ Agglutinin (WGA) staining (Sigma, USA). On the 28th day post‐surgery, mice were euthanized, and the hearts were paraffin‐embedded and sectioned in 10 µm slices. Masson's dye (Sigma, USA) was used to assess the extent of cardiac fibrosis. Echocardiographic assessments were performed at four‐time points: before surgery, on the 3rd day post‐surgery, on the 7th day post‐surgery, and 28th day post‐surgery.

### In Vivo Biodistribution and Targeting Specificity of PL720

To elucidate the cascade targeting effect of PL720 at different phases of MI/R, as shown in Figure S4a,b (Supporting Information), a single dose of PL720 was administered during the ischemia–reperfusion phase and the late reperfusion inflammatory phase, respectively.

During the ischemia–reperfusion phase (Figure S4a, Supporting Information), the mice were randomly divided into three groups: sham+ PBS, sham+ DiR‐labeled PL720, and MI/R+ DiR‐labeled PL720. Sham and MI/R mice were injected with PBS or DiR‐labeled PL720 immediately after surgery. After 6 h time point post‐surgery, a subset of mice from each group (*n* = 3) was euthanized and perfused with saline and paraformaldehyde to collect the brain, heart, lungs, liver, spleen, and kidneys. Near‐infrared fluorescence images of isolated organs were captured using an in vivo near‐infrared (NIR) fluorescence imaging system (IVIS Spectrum Imaging System, Caliper Life Sciences, USA) and quantitatively analyzed using Living Image 5.0. After 12 h time point post‐surgery, mice from each group (*n* = 3) were euthanized to obtain heart tissue samples. Transverse frozen sections were prepared at the injury site. CLSM was used to observe fluorescence and characterize the distribution of PL720 in the cardiac tissue.

During the late reperfusion inflammatory phase (Figure S4b, Supporting Information), mice were randomly divided into four groups: sham +PBS, sham+ DiR‐labeled PL720, MI/R+ DiR‐labeled PL720 62Pblock, and MI/R+ DiR‐labeled PL720. At 72 h time point post‐surgery, sham‐operated and MI/R mice were intravenously injected with PBS, DiR‐labeled PL720, or the DiR‐labeled PL720 62Pblock. At the 78‐h time point, a subset of mice from each group (*n* = 3) was euthanized. The brains, hearts, lungs, livers, spleens, and kidneys were collected after perfusion with saline and paraformaldehyde. NIR fluorescence images of the excised organs were captured and measured using an IVIS Spectrum Imaging System. At 84 h time point, mice from each group (*n* = 3) were euthanized to obtain heart tissue, and transverse frozen sections were prepared at the injury site. CLSM was used to observe the fluorescence and characterize the distribution of PL720 at the cardiac tissue level.

### RT‐PCR Assay

Inflammatory factor expression assays were performed using RT‐PCR. Total RNA was isolated from left ventricular tissue using TRIzol reagent (Sigma, USA). cDNA synthesis was performed using the PrimeScript RT Master Mix Kit (TaKaRa, China). RT‐PCR was performed using TB Green Premix Ex Taq Kit (TaKaRa) and an Mx3000p RT‐PCR system (Agilent, USA). Primers used in these experiments are listed in Table S1 (Supporting Information). The relative expression of the data was analyzed using the 2‐ΔΔCt method.

### Western Blot Analysis

Protein samples were obtained from left ventricular tissues and cells using protein extraction reagents. Protein concentration was quantified using a BCA protein assay kit. The protein samples were electrophoresed on 8% SDS‐PAGE gels and transferred to PVDF membranes. After blocking the membranes with 5% skim milk at room temperature (25 °C) for 2 h, primary antibodies against BAX (Abcam, USA), BCL‐2 (Abcam, USA), AKT (Abcam, USA), p‐AKT (Abcam, USA), STAT3 (Abcam, USA), p‐STAT3 (Abcam, USA), iNOS (Abcam, USA), and CD206 (Abcam, USA) were incubated overnight at 4 °C. Following three washes with Tris‐buffered saline containing Tween 20 (TBST), the membranes were incubated with secondary antibodies for another 2 h. Glyceraldehyde‐3‐phosphate dehydrogenase (GAPDH) was used as a housekeeping gene. Finally, the membranes were developed using chemiluminescent reagents, and the protein bands were quantitatively analyzed using the ImageJ software.

### Echocardiogram

Cardiac function within each group was evaluated using 2D‐guided M‐mode echocardiography (Vevo 2100, Canada), focusing on the structural and functional attributes of the hearts of the mice. In brief, the mice were continuously anesthetized with 1–2% isoflurane. The chest area of the mice was shaved, and the parasternal long‐axis view was obtained to measure various parameters: EF, FS, LVID; d, LVID; s, lLV Vol; d, and LV Vol; s.

### Statistical Analysis

Data are presented as mean ± standard error. Student's *t*‐test was used to assess significant differences between two groups. For comparisons among multiple groups, analysis of variance (ANOVA) was conducted, followed by Tukey's post hoc test for correction. Statistical significance was defined as a *p*‐value of less than 0.05. Data analysis was performed using GraphPad Prism 7.0 (GraphPad Software, USA).

## Conflict of Interest

The authors declare no conflict of interest.

## Supporting information

Supporting Information

## Data Availability

The data that support the findings of this study are available from the corresponding author upon reasonable request.
