# Supplementary material for: Platelet Membrane Nanocarriers Cascade Targeting Delivery System to Improve Myocardial Remodeling Post Myocardial Ischemia–Reperfusion Injury
Source: Adv Sci (Weinh). 2024 Feb 12;11(16):2308727. doi: 10.1002/advs.202308727 (PMC11040350; doi:10.1002/advs.202308727)
Supplement: Supplementary file 1 — Supporting Information [file ADVS-11-2308727-s001.pdf]

ADVANCED SCIENCE
Open Access

Supporting Information

for *Adv. Sci.*, DOI 10.1002/advs.202308727

Platelet Membrane Nanocarriers Cascade Targeting Delivery System to Improve Myocardial Remodeling Post Myocardial Ischemia–Reperfusion Injury

Xuan Xu, Mingxi Li, Fuchao Yu, Qin Wei, Yang Liu, Jiayi Tong and Fang Yang**

Supporting Information

Platelet Membrane Nanocarriers Cascade Targeting Delivery System to Improve Myocardial Remodeling Post Myocardial Ischemia - Reperfusion Injury

Xuan Xu[#], *Mingxi Li*[#], *Fuchao Yu, Qin Wei, Yang Liu, Jiayi Tong*[*], *and Fang Yang*[*]

X. Xu, F. Yu, Q. Wei, J. Tong

Department of Cardiology, Zhongda Hospital Affiliated to Southeast University, 87 Dingjiaqiao, Nanjing, P.R China 210009

M. Li, Y. Liu, F. Yang

State Key Laboratory of Digital Medical Engineering, Jiangsu Key Laboratory for Biomaterials and Devices, School of Biological Sciences and Medical Engineering, Southeast University, Nanjing 210096, P. R. China.

[#] These authors contributed equally: Xuan Xu, Mingxi Li

[*]Corresponding authors. E-mail: 101007925@seu.edu.cn (Jiayi Tong); yangfang2080@seu.edu.cn (Fang Yang)

Supplementary methods

Cytotoxicity PL720: Cytotoxicity was assessed by treating THP-1 cells, HUVEC, H9C2, and BMDM with PL720. Cell viability was evaluated using a CCK-8 kit (Beyotime, China) according to the manufacturer's instructions. Briefly, cells were seeded at a density of 5×10^4 cells/ well in 96-well plates. Upon reaching 80% confluency for HUVECs and H9C2 cells,

and a density of 1×10^5 for THP-1 cells, the cells were exposed to 1 μL of PL720, PNV, or FTY720 (100 nM) for 12 h. After CCK8 treatment, the absorbance was measured at 450 nm using a Multiskan SkyHigh microplate reader (Thermo Scientific, USA).

Biosafety assessment of PL720: On the day following treatment with two doses of PL720 (the 4th day after surgery), blood samples were collected from mice in each group through orbital bleeding to assess their coagulation function. Plasma was collected for the assessment of coagulation parameters (PT and APTT) following the manufacturer's instructions.

On the third day after the administration of two doses of PL720 (postoperative day six), blood samples were acquired from mice in each group through orbital blood sampling, followed by serum collection. Hepatic function markers, including aspartate aminotransferase (ALT), alanine aminotransferase (AST), and γ-glutamyl transpeptidase (γ-GT), were assessed alongside renal function indicators, namely urea nitrogen (UREA) and creatinine (CREA), using kits from Kayto (China). Furthermore, on the25th day after the second dose of PL720 treatment (28th day post-surgery), histopathological changes were evaluated by H&E staining of the brain, lung, liver, spleen, and kidney.

PL720 targeting to injured blood vessel experiment: To investigate the targeting effect of PL720 on injured blood vessels, the aorta from 10-week-old SD rats was obtained, and the endothelial layer was gently scraped using a scalpel. The injured or normal control aortas were treated with a 60 s rinse of DiI-labeled PL720, followed by washing with PBS for 3 times. PL720 adhesion to the aorta was examined using a fluorescence microscope.

Supplementary Figures

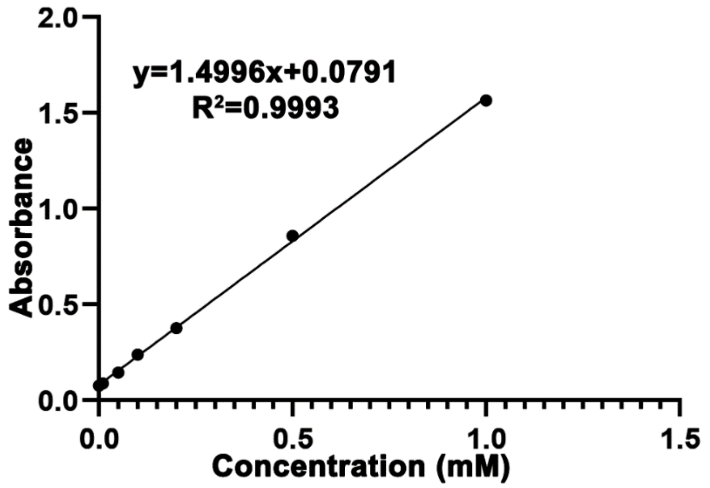

Figure S1. The purified L-arginine standard curve determined from the corresponding absorbance *vs.* L-arginine concentration.

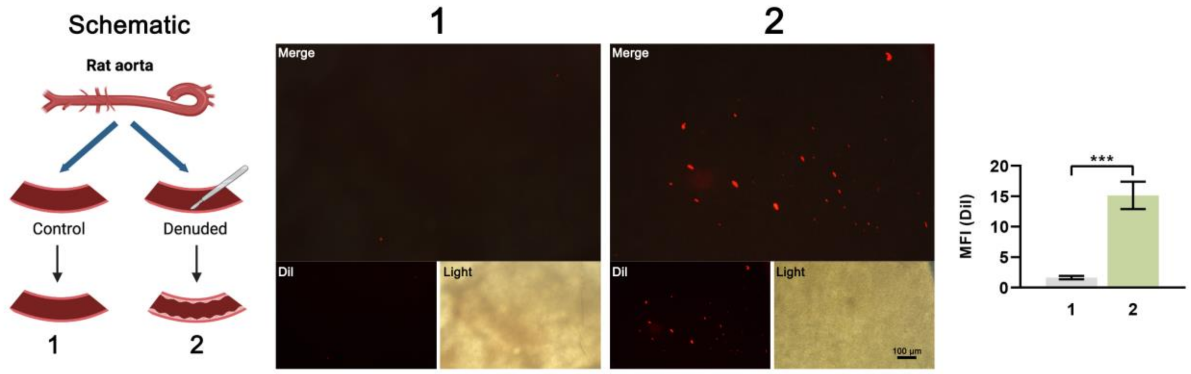

Figure S2. A schematic diagram showing the experimental design for denuded rat aorta binding. Representative fluorescent micrographs showing the adherence of DiI-labelled PL720 on control or denuded aortas (n=3). Results are reported as mean ± SD. Data were analyzed using one-way ANOVA followed by two-tailed Student's t test. ns indicates non-significant ($P > 0.05$). * $P < 0.05$, **$P < 0.01$, and ***$P < 0.001$.

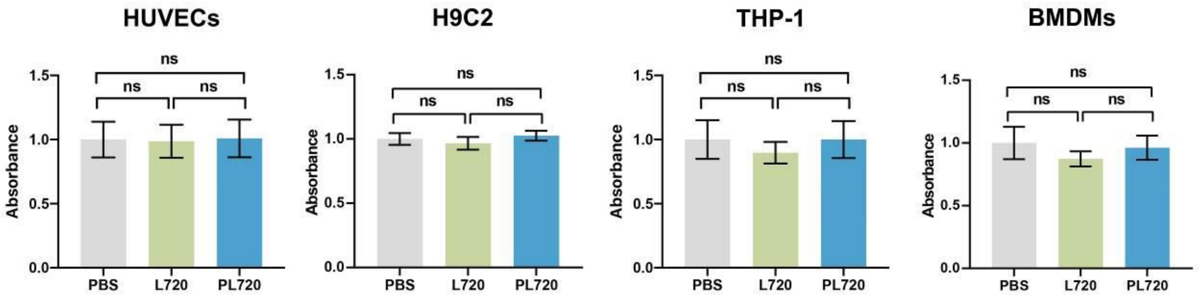

Figure S3. HUVECs, H9C2, THP-1, and BMDMs viability measured by CCK8assay (n = 3). Results are reported as mean ± SD. Data were analyzed using one-way ANOVA followed by two-tailed Student's t test. ns indicates non-significant (P > 0.05). * P < 0.05, **P < 0.01, and ***P < 0.001.

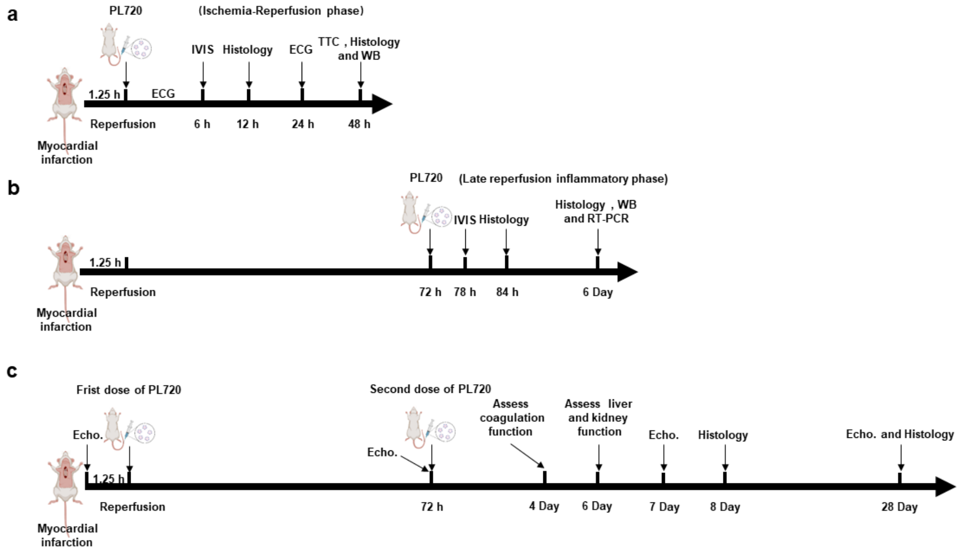

Figure S4. a. Experimental protocol for the reperfusion phase treatment. b. Experimental protocol for the inflammation phase treatment. c. Experimental protocol for the two doses treatment.

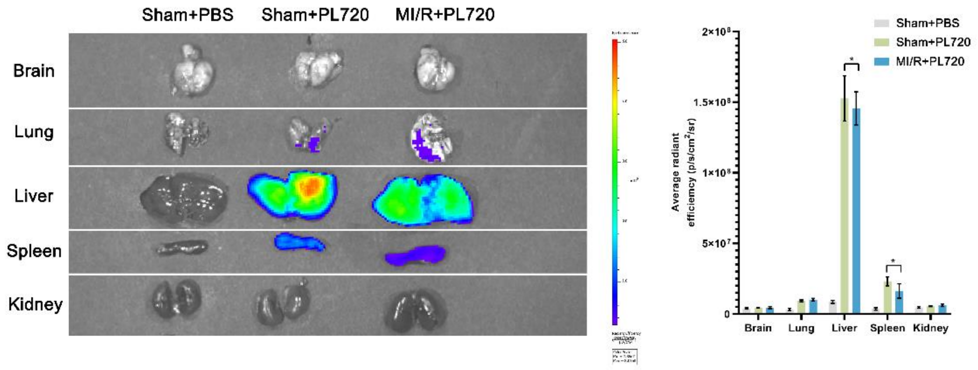

Figure S5. NIR images of major organs of sham or MI/R mice after treated with DiR labeled PL720, and quantitative analysis of the accumulation of PL720 (n = 3). Results are reported as mean ± SD. Data were analyzed using one-way ANOVA followed by two-tailed Student's t test. ns indicates non-significant ($P > 0.05$). * $P < 0.05$, **$P < 0.01$, and ***$P < 0.001$.

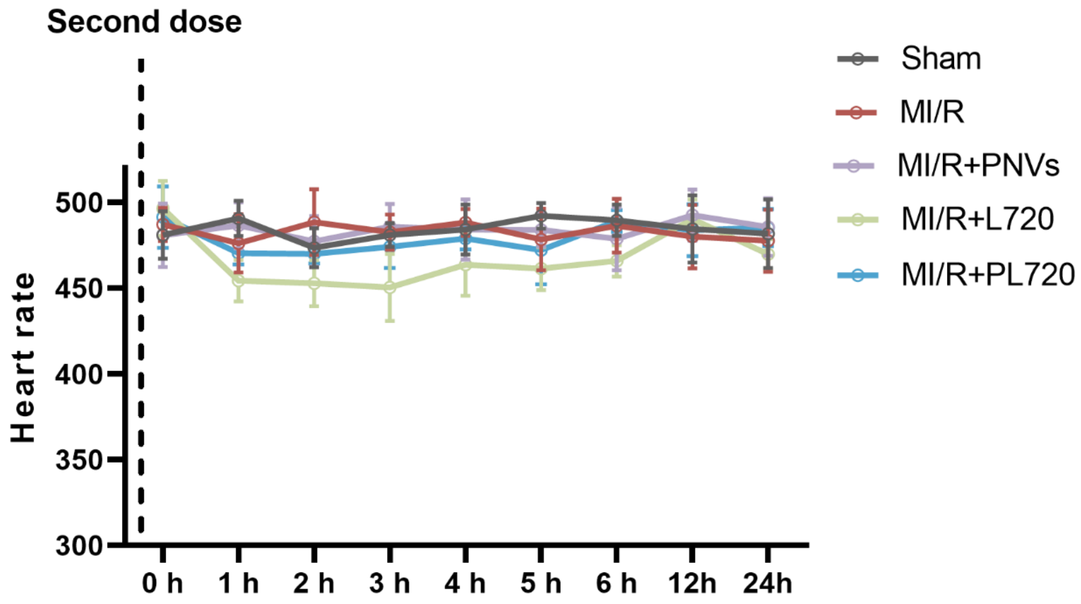

Figure S6. Heart rates of mice in each group at different times (0, 1, 2, 3, 4, 5, 6,12 and 24 h) after the second injection of PBS, PNVs, L720 and PL720 (n=6).

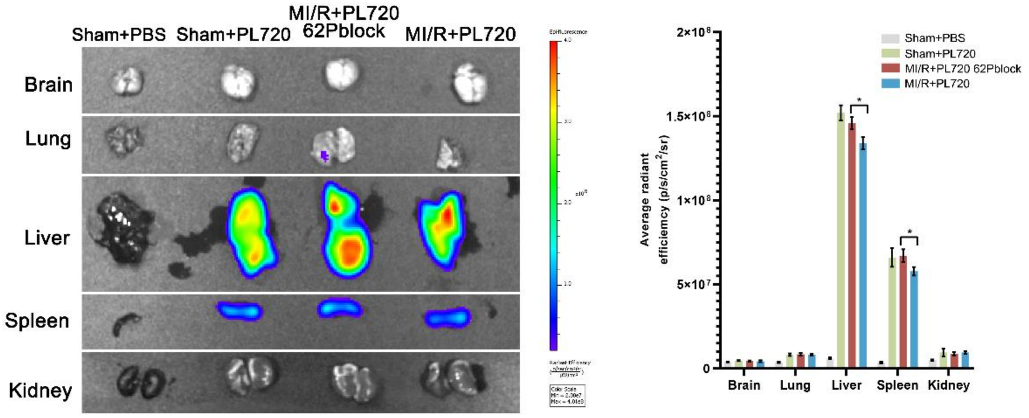

Figure S7. NIR images of major organs of sham or MI/R mice after treated with DiR labeled PL720 and PL720 62Pblock, and quantitative analysis of the accumulation of PL720 (n = 3). Results are reported as mean ± SD. Data were analyzed using one-way ANOVA followed by two-tailed Student's t test. ns indicates non-significant (P > 0.05). * P < 0.05, **P < 0.01, and ***P < 0.001.

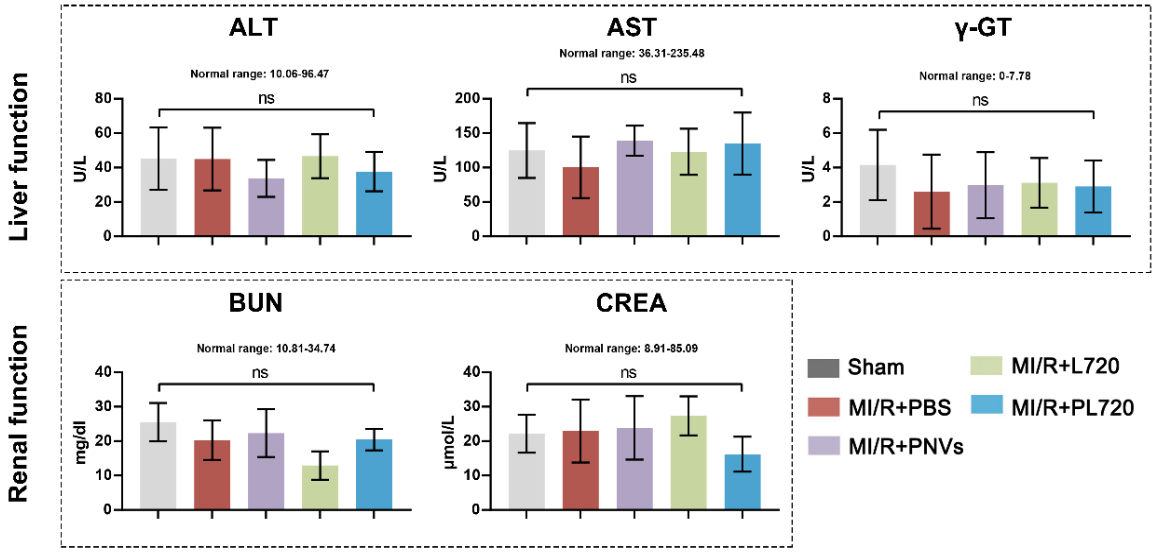

Figure S8. Liver and renal function of mice after treated by PBS, PNVs, L720, and PL720, respectively (n = 5). Results are reported as mean ± SD. Data were analyzed using one-way ANOVA followed by two-tailed Student's t test. ns indicates non-significant (P > 0.05). * P < 0.05, **P < 0.01, and ***P < 0.001.

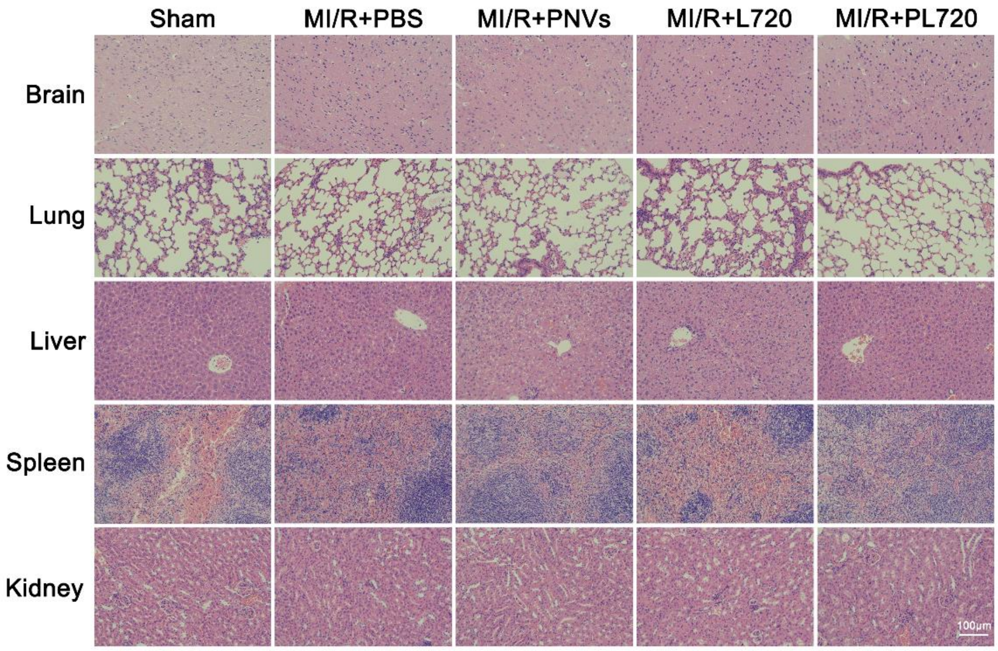

Figure S9. Histological characteristics of major organs (brain, lung, liver, spleen, and kidney) were detected by H&E staining.

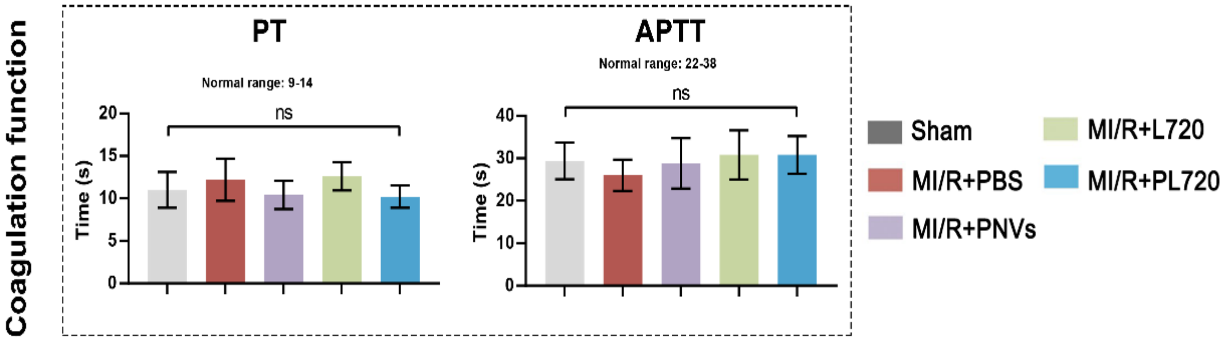

Figure S10. Coagulation function of mice after treated by PBS, PNVs, L720, and PL720, respectively (n = 5). Results are reported as mean ± SD. Data were analyzed using one-way ANOVA followed by two-tailed Student's t test. ns indicates non-significant ($P > 0.05$). * $P < 0.05$, **$P < 0.01$, and ***$P < 0.001$.

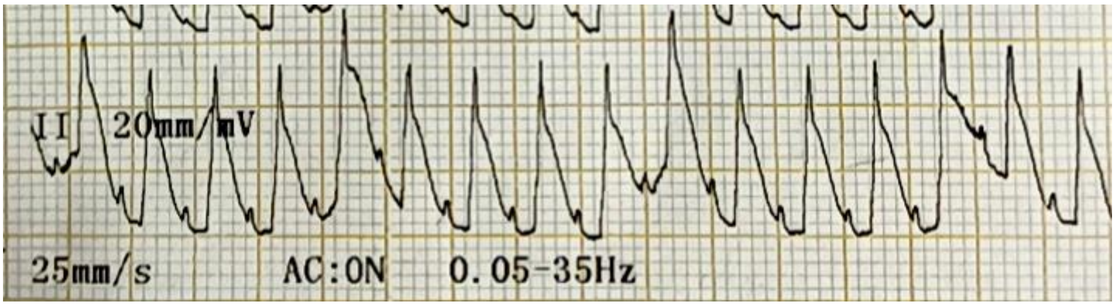

Figure S11. Electrocardiograms of mice after coronary artery ligation.

Table S1. For gene expression quantification, cDNA was synthesized using PrimeScript™ RT Master Mix Kit (TaKaRa, China) and then the PCR process was performed by TB Green Premix Ex Taq Kit (TaKaRa). β- actin was used as internal control.

Gene	Forward Primer (5' to 3')	Reverse Primer (5' to 3')
mmu-β-Actin	TGAGCTGCGTTTTACACCCT	GCCTTCACCGTTCCAGTTTT
mmu-IL-1β	GAAATGCCACCTTT TGACAGTG	TGGATGCTCTCATCAGGACAG
mmu-Tnf-α	TAGCCCACGTCGTAGCAAAC	GCAGCCTTGTCCCTTGAAGA
mmu-IL-10	GGCAGAGAACCATGGCCCAGAA	AATCGATGACAGCGCCTCAGCC
mmu-TGF-β	TGCGCTTGCAGAGATTAAAA	CGTCAAAAGACAGCCACTCA
